# Oncogenic DMTF1β promotes cancer cell motility by regulating autophagy through ULK1 stabilization

**DOI:** 10.1002/1878-0261.70275

**Published:** 2026-05-26

**Authors:** Jun Xu, Nicolas J. Niklaus, Anna M. Schläfli, Igor Tokarchuk, Magali Humbert, Federico La Manna, Bich Vu, David Maul, Ramin Radpour, Marianna Kruithof‐de Julio, Inti Zlobec, Jörn Dengjel, Bruce E. Torbett, Mario P. Tschan

**Affiliations:** ^1^ Institute of Tissue Medicine and Pathology, Division of Experimental Pathology University of Bern Switzerland; ^2^ Graduate School for Cellular and Biomedical Sciences University of Bern Switzerland; ^3^ Department of Biomedical Research, Division of Urology University of Bern Switzerland; ^4^ Department of Biology University of Fribourg Switzerland; ^5^ Department of Biomedical Research, Division of Tumor immunology University of Bern Switzerland; ^6^ Department of Medical Oncology, Inselspital, Bern University Hospital University of Bern Switzerland; ^7^ Department of Immunology and Microbiology The Scripps Research Institute La Jolla CA USA; ^8^ Department of Pediatrics, School of Medicine University of Washington Seattle WA USA; ^9^ Center for Immunity and Immunotherapies Seattle Children's Research Institute Seattle WA USA

**Keywords:** autophagy, cell migration, DMTF1, oncogene, splice variant, ULK1

## Abstract

The cyclin D binding Myb‐like Transcription Factor 1 (DMTF1) is a haploinsufficient tumor suppressor in various tumors. Alternative splicing generates a dominant negative, truncated version of full‐length DMTF1α, named DMTF1β. DMTF1β has so far been described as an oncogene in breast cancer development. However, a clear understanding of how DMTF1β contributes to carcinogenesis remains unknown. Analyzing DMTF1β protein expression in breast cancer cell lines, as well as a highly metastatic prostate cancer cell line, confirmed a positive correlation between DMTF1β expression and tumorigenic potential. Specifically, knocking down DMTF1β in aggressive MDA‐MB‐231 breast and PC3MPRO4 prostate cancer cells significantly reduced wound closure and tissue invasion. β‐specific interactome and RNA‐sequencing studies in DMTF1β overexpressing and knockdown cells, respectively, suggest that DMTF1β expression is associated with the autophagy recycling pathway. Depleting DMTF1β levels in cancer cells significantly decreased autophagic flux. Moreover, inhibiting autophagy led to decreased migration of DMTF1β expressing breast cancer cells. Mechanistically, DMTF1β protein interacts with and stabilizes the key autophagy protein ULK1. In conclusion, we identified a novel function for the alternatively spliced DMTF1 gene product in autophagy‐dependent cancer cell motility.

## Graphical abstract

In the current study, we demonstrate that the oncogene DMTF1β regulates ULK1 stability by reducing its proteasomal degradation in cancer cells. This stabilization enables ULK1 to induce autophagy, which in turn facilitates cancer cell migration. Consequently, reduced DMTF1β levels lead to decreased autophagy and impaired cancer cell migration.

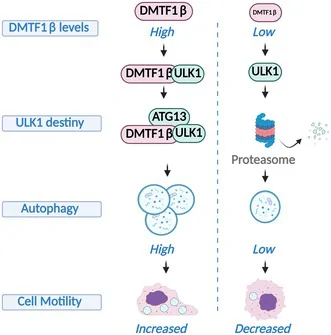

**Abbreviations**
DMTF1: the cyclin D binding Myb‐like Transcription Factor 1
ECM: extracellular matrix
EMT: epithelial–mesenchymal transition
ER: estrogen receptor
FDR: false discovery rate
GSEA: gene set enrichment analysis
HDR: homology‐directed repair
LIR: LC3‐interacting region
LLPDA: long‐lived protein degradation assay
MWU: Mann–Whitney U
NES: normalized enrichment score
PCA: principal component analysis
PLA: proximity ligation assay
PR: progesterone receptor
RNA‐seq: RNA sequencing
RNP: ribonucleoprotein
TNBC: triple‐negative breast cancer
TWA: two‐way ANOVA

## 1 Introduction

The DMTF1 transcription factor regulates an array of downstream targets that are involved in cell cycle arrest and apoptosis. Its canonical function is the transactivation of the *CDKN2A/p14* ^*ARF*^ gene in a homodimeric fashion [1, 2, 3, 4]. The tumor suppressor p14^ARF^ sequesters the ubiquitin protein ligase MDM2 to nucleoli, thereby preventing degradation of the master tumor suppressor p53. Thus, by positively regulating p14^ARF^, DMTF1 stabilizes p53, which in turn can induce, among others, cell cycle arrest and apoptosis. In addition, DMTF1 protein can directly heterodimerize with p53 to transactivate the proapoptotic BCL2 family member BBC3/PUMA, thereby facilitating intrinsic apoptosis [5, 6, 7, 8]. Interestingly, alternative splicing of the DMTF1 pre‐mRNA yields, in addition to the full‐length DMTF1 (hereafter referred to as DMTF1α), two truncated isoforms named DMTF1β and DMTF1γ, respectively. These isoforms arise from two alternative 3′ splice sites that lead to partial insertions within intron 9, resulting in a reading frame shift and premature stop codons. The resulting alternatively spliced pre‐mRNAs encode for two smaller proteins, each containing a short isoform‐specific domain [4, 9]. Interestingly, DMTF1β and, to a lesser degree, DMTF1γ negatively regulate DMTF1α. Mammary tumors often express increased DMTF1β levels as compared to surrounding healthy tissue, and increased DMTF1β levels accelerate carcinogenesis in mice [10]. In addition, an increased DMTF1β/α ratio is associated with reduced macrophage differentiation, a reduced overall survival of breast cancer patients, as well as increased chemotherapy resistance of breast cancer cells [4, 9, 10, 11, 12, 13]. Thus, DMTF1β is considered an oncogenic counterpart of DMTF1α with an important role in breast cancer [10, 11].

Breast cancer is the most common diagnosed cancer type among women, whereas prostate cancer is the most abundant tumor‐related malignancy in men [14, 15]. The short‐term survival rate of early and localized breast and prostate cancers is high with more than 90% overall survival. However, survival drops to 30% in advanced cases with invasive tumors. In addition, patients who survived the initial disease course frequently experience highly aggressive recurrences accompanied with formation of metastatic lesions that are the main cause of death [14, 16, 17]. Hence, there is an urgent need to better understand the metastatic mechanism(s) of breast and prostate cancers.

Autophagy is a catabolic process responsible for the degradation of cytoplasmic constituents, such as damaged or superfluous organelles or proteins in a lysosome‐dependent manner. A key function of autophagy is quality control and maintenance of cellular homeostasis. Autophagy is activated upon diverse stress stimuli as an adaptive survival response. On a molecular level, macroautophagy, referred to as autophagy from here on, is a tightly regulated pathway that depends on core autophagy‐related (ATG) genes that function in complexes at distinct steps of the process. Autophagy starts with the formation of a double membrane structure called the phagophore under the control of the ULK1 and Beclin1 protein complexes. Next, the phagophore elongates, engulfs cargo and closes to form the so‐called autophagosome. This process is controlled by two ubiquitin‐like conjugation steps. The first one serves to generate an E3 ligase complex known as the ATG5‐ATG12‐ATG16L complex. This E3 ligase further directs ATG8 lipidation, allowing for insertion of ATG8s into autophagosome membranes. After closure, the autophagosome fuses with a lysosome where the content is degraded via lysosomal hydrolases. The degraded content yields cellular building blocks, such as amino acids, glucose, and free fatty acids, that can be recycled for macromolecule synthesis [18, 19, 20, 21].

In our study, we investigated the oncogenic role of DMTF1β in breast and prostate cancer cells. Our findings indicate that the oncogenic DMTF1β isoform is associated with a more mesenchymal phenotype. We identified the key autophagy protein ULK1 as a novel DMTF1β interaction partner, with DMTF1β supporting ULK1 protein stability and autophagy activity. Taken together, our studies have identified novel oncogenic functions of DMTF1β in regulating cancer cell motility by increasing autophagic activity.

## 2 Material and methods

### 2.1 Cell lines and culture conditions

The HEK293T (RRID: CVCL_0063) cell line was obtained from ATCC and cultured in Dulbecco's Modified Eagle's Medium (DMEM, Sigma‐Aldrich/Merck, Buchs, Switzerland), complemented with 5% FBS and 1% HEPES (Sigma‐Aldrich/Merck). The cell line was incubated at 37 °C in a humidified 7.5% CO_2_ atmosphere. The human breast cancer cell line MCF7 (RRID: CVCL_0031) was obtained from American Type Culture Collection (ATCC) and cultured in DMEM (Sigma‐Aldrich/Merck), complemented with 10% FBS. The human breast cancer cell line MDA‐MB‐231 (RRID: CVCL_0062) was kindly provided by C. Lengerke and H.U. Simon and cultured in Roswell Park Memorial Institute Medium (RPMI‐1640, Sigma‐Aldrich/Merck), complemented with 10% FBS. The metastatic subline PC‐3‐M‐PRO4 (RRID: CVCL_D579) is derived from the human prostate cancer cell line PC‐3 (RRID: CVCL_0035), which was kindly provided by M. Kruithof‐de Julio. These cells were cultured in Dulbecco's modified Eagle's medium (DMEM), complemented with 10% FBS. All cancer cell lines were incubated at 37 °C in a humidified 5% CO_2_ atmosphere. All experiments were carried out with Mycoplasma‐free cells.

All cell lines were authenticated within the past three years. Authentication was performed by Microsynth AG using highly polymorphic short tandem repeat (STR) markers. Allele calling and data interpretation were conducted with GeneMarker HID, and the resulting profiles were compared to the reference STR profiles of each cell line in Cellosaurus.

### 2.2 Transfection, lentiviral transduction, and CRISPR‐Cas9‐mediated knockout

HEK293T cells were transiently transfected with the desired construct and lentiviral packaging plasmids (pMD2G VSGV, pMDlg/pRRE, and pRSV‐rev) via calcium‐phosphate precipitation. Following constructs were used in this study: lentiGuide‐DMTF1(1) (gRNA: GAAACTTCCTCGTTACCCA), lentiGuide‐DMTF1(2) (gRNA: GTGCAACAACTGATATGCA), pLV‐EF1α‐IRES‐Hyg, pLV‐EF1α‐Flag‐DMTF1β‐IRES‐Hyg, pLV‐EF1α‐Flag‐DMTF1ΔMHR‐IRES‐Hyg, pLKO.1‐Hyg (non‐mammalian shRNA control), pLV‐U6‐shDMTF1β_1‐EF1α‐IRES‐Hyg (shRNA: AGTCAAATGGGAAGAAGAA), pLV‐U6‐shDMTF1β_2‐EF1α‐IRES‐Hyg (shRNA: ACTTTGGCTCTCAAAGTATTG), shULK1 (NM_003565.x‐535s1c1) and shVPS34 (NM_002647.2‐930s1c1). 72 h after transfection, the supernatants were collected and filtered through a 0.45 μm nitrocellulose membrane.

The cells were infected with the virus supplemented with 8 μg/mL Polybrene prior to antibiotic selection. Cell lines transduced with lentiGuide for DMTF1 were transiently transfected with lentiCas9‐Blast (#52962; Addgene, Watertown, MA, USA) via calcium‐phosphate precipitation and were selected for single clones.

siRNA targeting p53 was transiently transfected into MCF7 cells via Lipofection2000 according to the manufacturer's protocol. Following siRNA were used: siScramble: 5′‐AGGUAGUGUAAUCGCCUUGTT‐3′ (Microsynth), sip53_1: 5′‐AUUUGCGUGUGGAGUAUUUTT‐3′ (Microsynth), sip53_2: 5′‐GACUCCAGUGGUAAUCUACTT‐3′ (Microsynth).

### 2.3 Generating HiBiT knock‐in cells with genome editing

Cas9 recombinant protein, together with sgRNA, single‐stranded oligodeoxynucleotide (ssODN) donor template and the HDR enhancer, was purchased from Integrated DNA Technologies (IDT). The following sequences were used: sgRNA: AGAGTCTGCCAATCAGCATT, ssODN: TATTGCTGCCCACAACTTCCAAACCAGTCAAATGGGAAGAAGAAGAATGAAGAAGTCTCCGTGAGCGGCTGGCGGCTGTTCAAGAAGATTAGCTAAATGATGAAATTAAAAGGGAGAAAACAATTGAATTAAATGCTGATTGGCAGACTCTTGTTT. During electroporation, a 5 : 1 ratio of sgRNA to Cas9 was maintained, with each reaction containing 15 pmol of Cas9 protein. RNP complexes were formed by incubating Cas9 and sgRNA in buffer R (from the Neon electroporation kit by Thermo Fisher Scientific, Basel, Switzerland) at room temperature for 15 min. Following this, 1 μL of 60 μm ssODN was introduced into the mixture. After washing the cells with PBS, 50 000 cells were resuspended in buffer R. The RNP/ssODN mixture was then added to the cells, and electroporation was carried out using the Neon electroporation kit, applying two pulses at 1250 V with a 20 ms pulse width and a 10 μL tip. Ninety‐six hours later, a limiting dilution assay was performed to isolate single clones, which were analyzed for HiBiT tag integration using a lytic detection system as per the manufacturer's instructions.

### 2.4 Immunoblotting

For immunoblotting, cells were lysed in urea buffer consisting of 8 M urea and 0.5% (v/v) Triton X‐100 supplemented with Complete™ Protease Inhibitor Cocktail (Roche, Basel, Switzerland) and PhosSTOP™ (Roche). Protein concentration was determined using Bradford Assay (Bio‐Rad Laboratories, Cressier, Switzerland). Proteins were separated by SDS/PAGE and transferred onto PVDF membranes using the ChemiDoc Imaging System (Bio‐Rad Laboratories). The immunoblots were blocked with 5% non‐fat milk in TBS‐T followed by incubation with the primary antibodies and HRP‐conjugated secondary antibody. The bands were detected using Clarity™ Western ECL Substrate (Bio‐Rad Laboratories) and on the ChemiDoc detection system (Bio‐Rad Laboratories).

The following primary antibodies were used: anti‐DMTF1 (GTX112228; GeneTex, Irvine, CA, USA), anti‐DMTF1 (ab87782; Abcam, Cambridge, UK), anti‐cleaved PARP (9541; Cell Signaling Technology, Leiden, The Netherlands), anti‐LC3B (NB600‐1384; Novus Biologicals, Abingdon, UK), anti‐p53 (p6874; Sigma‐Aldrich, Buchs, Switzerland), anti‐ULK1 (4773S; Cell Signaling Technology), anti‐ATG13 (13 273; Cell Signaling Technology), anti‐VPS34 (NB110‐87320SS; Novus Biologicals), anti‐Beclin1 (3738S; Cell Signaling Technology), anti‐Flag®M2 (F3165; Sigma‐Aldrich). The following secondary antibodies were used: anti‐rabbit IgG HRP‐linked (7074 s; Cell Signaling Technology), anti‐mouse IgG HRP‐linked (7076 s; Cell Signaling Technology).

### 2.5 Flow cytometry

Cells were trypsinized and resuspended in media. Next, cells were washed in cold PBS supplemented with 1% FBS and then incubated 1:200 in CD24‐PE/Cy7 (311 120; BioLegend, Amsterdam, The Netherlands) and 1:200 in CD44‐PB (103 020; BioLegend) for 20 min on ice in the dark. After incubation, cells were washed and resuspended in ice‐cold FACS buffer. Data acquisition was carried out on a FACS LSR‐II (BD Biosciences, Allschwil, Switzerland) and analyzed using FlowJo software.

### 2.6 Wound closure assay

2–3 × 10^5^ cells were plated into 24‐well plates. After adherence, a scratch was applied across the well with a 200 μL pipette tip; cells were washed twice with medium and monitored up to 24 h using the Cell‐IQ® cell imaging and analysis system (Chip‐Man Technologies, Finland) and analyzed with the Cell Activation software.

### 2.7 Trans‐well migration and invasion assay

Cells were serum starved 24 h prior to the experiment. Next, cells were detached with Accutase (Sigma‐Aldrich) and resuspended in serum‐free media. In a separate 24‐well plate, 600 μL of media containing 20% FBS was added to the wells, and 8 μm pore sized trans‐well inserts (Sarstedt) pre‐coated with or without Matrigel were placed into the wells. 1 × 10^5^ cells were seeded on top of the inserts. After indicated times, inserts were fixed with 4% PFA and stained with crystal violet. After staining, cells were washed 3 times with water, and cells on top of the insert were removed with a cotton tip. Inserts were imaged with an EVOS FL imaging system (Fisher Scientific, Reinach, Switzerland) and analyzed by imagej software.

### 2.8 Reverse transcription PCR and quantitative PCR

Total RNA was extracted using the GenElute™ Total RNA Purification (Sigma‐Aldrich/Merck) Kit according to the manufacturer's protocol. Total RNA was reverse transcribed using ReadyScript® cDNA Synthesis Mix (Sigma‐Aldrich/Merck). Standard polymerase chain reaction (PCR) was performed using JumpStart™ Taq‐Polymerase (Sigma‐Aldrich/Merck) according to the manufacturer's protocol. The following primers were used for detection of DMTF1 isoforms PCR: DMTF1α, β and γ (Fwd: 5′‐TACAGGACTATAGCATGGGGTC‐3′, Rev: 5′‐ACTTCCCTGTGTTGCAAGTATC‐3′), DMTF1β and γ (Fwd: 5′‐TACAGGACTATAGCATGGGGTC‐3′, Rev: 5′‐CCATTTGA CTGGTTGGAAGTTG‐3′), 18 s rRNA (Fwd: 5′‐TCGAGGCCCTGTAATTGGAA‐3′, Rev: 5′‐CTTTAATATACGCTATTGGAGCTGGAA‐3′).

The TaqMan® Fast Universal PCR Master Mix (2×), no AmpErase® UNG (Fisher Scientific) together with the StepOnePlus (Applied Biosystems, Basel, Switzerland) qPCR system were used according to the manufacturer's protocol to perform quantitative PCR. The following gene expression assays were used during this project: HMBS (Fwd: 5′‐GGCAATGCGGCTGCAA‐3′, Rev: 5′‐GGGTACCCACGCGAATCAC‐3′, Probe: 5′‐CTCATCTTTGGGCTGTTTTCTTCCGCCT‐3′), DMTF1α (Fwd: 5′‐CCATGTGGGAAAATATACACCTGAA‐3′, Rev 5′‐CCCTATTGTTGCCCAGTCATTG‐3′, Probe 5′‐AGAAGCTCAAGGAGCTCCGGATAAAGCAT‐3′), DMTF1β (Fwd: 5′‐ATTGAGAAGCTCAAGGAACAACTGT‐3′, Rev: 5′‐CCATTTGACTGGTTTGGAAGTTG‐3′, Probe: 5′‐CCACACTTTCAAACTTTGGCTCTCAAAGTATTGC‐3′), CDH1 (Forward 5′‐GAACAGCACGTACACAGCCCT‐3′, Reverse 5′‐GCAGAAGTGTCCCTGTTCCAG‐3′, Probe 5′‐ATCATAGCTACAGACAATGGTTCTCCAGTTGCT‐3′), WIPI1 (Hs00215872_m1; ThermoFisher) and VPS34 (Hs00176908_m1; ThermoFisher).

### 2.9 Immunofluorescence microscopy

Cells were grown on sterile coverslips and fixed for a few seconds in 4% PFA before fixation and permeabilization in ice‐cold methanol. Fixed cells were blocked in PBS/1% BSA/0.1% Tween followed by primary and secondary antibody incubation at RT for 1 h. Thereafter, cells were washed and mounted in SlowFade™ Gold Antifade Mountant with DAPI (Thermofisher). Data acquisition was carried out on an Olympus IX81 (Olympus Life Science) using a 63× oil objective and images were analyzed and quantified using the imagej software.

The following antibodies were used in this project: anti‐LC3B (3868; Cell Signaling Technology), Cy3 anti‐Rabbit IgG (H + L) (111–166‐045; Jackson ImmunoResearch/LuBioScience, Zurich, Switzerland).

### 2.10 Long‐lived protein degradation assay (LLPDA)

Cells were seeded depending on the cell line at a density between 2–5 × 10^4^/mL. 200 μL 0.2 μCi of ^14^C‐Valin/mL (Amersham) was added to each well after the cells adhered. Cells were incubated for 3 days, washed twice with media and incubated for 1 h in the presence of 10 mm non‐radioactive L‐Valin (Sigma‐Aldrich/Merck) before the 24 h chase phase was started in the presence or absence of VPS34IN1 (S7980; Selleck Chemicals LLC/LuBioScience, Zurich, Switzerland) or Bafilomycin A1 (S1413, Selleck Chemicals LLC). Cells were lysed with 0.2 m NaOH (Merck, Darmstadt, Germany) and protein precipitations were carried out using 10% Trichloracetic acid (Sigma‐Aldrich/Merck). After incubation at 4 °C for 2 h, samples were centrifuged at 600 rcf for 20 min. Microscint‐40 (PerkinElmer, Schwerzenbach, Switzerland) was added to the supernatant and the pellet fractions and radioactivity were determined using a Microbeta2 instrument (PerkinElmer). Proteolysis was calculated as the percentage of radioactivity in the supernatant.

### 2.11 Zebrafish xenograft experiment

The zebrafish (ZF) transgenic lines Tg(fli1:GFP) were used for the *in vivo* studies of the prostate cancer cell lines. Zebrafish and embryos were raised, staged, and maintained according to standard procedures in compliance with the local animal welfare regulations and the EU Animal Protection Directive 2010/63/EU. 0.2 mm N‐phenylthiourea (Sigma‐Aldrich/Merck) was applied to prevent pigment formation from the first day's post‐fertilization (dpf). ZF xenograft experiments have been described in detail previously [22]. Briefly, two‐dpf ZF embryos were anesthetized with 0.003% tricaine (Sigma‐Aldrich/Merck) and positioned on a 10 cm petri dish coated with 1% agarose (Sigma‐Aldrich/Merck). Then 50–400 manually counted cells were injected into the duct of Cuvier using a Pneumatic Pico pump and a micromanipulator (WPI, Sarasota, FL, USA). After implantation with cancer cells, the ZF embryos (including non‐implanted controls) were maintained at 34 °C as a compromise between the optimal temperature requirements for fish and mammalian cells. Fluorescent image acquisition was performed using a Leica MZ16FA stereo microscope (Leica Microsystems GmbH, Wetzlar, Germany). Separate images of the various segments of the ZF embryos were blended to form a composite image using Adobe Photoshop CS6 software (Adobe Systems, Mountainview, CA, USA).

### 2.12 Proximity ligation assay (PLA)

Cells were grown on poly‐L‐Lysine‐treated 15 × 15 mm glass cover slides overnight before PLA was performed using the Duolink® Kit (Sigma‐Aldrich/Merck). Cells were washed in PBS, fixed with 100% ice‐cold Methanol for 10 min, before blocking with Duolink® blocking solution at 37 °C for 1 h. Thereafter, slides were incubated 1 h at RT with anti‐ULK1 (4773, Cell Signaling Technology, 1:200 or A7481, Sigma‐Aldrich 1:200), and anti‐HiBiT (N7200, Promega, Dübendorf, Switzerland, 1:600) antibodies at RT. From here on, the protocol was followed as described in the manufacturer's description. After washing, slides were dried and mounted in ProLong™ Gold Antifade Mountant with DAPI (Invitrogen, Basel, Switzerland). Z‐stack pictures were taken using an Olympus FluoView‐1000 confocal microscope with a 63× objective. Quantification of dots and image preparation was done using imagej software.

### 2.13 Next‐generation RNA sequencing (RNA‐seq)

Total RNA was extracted using the GenElute™ Total RNA Purification Kit according to the manufacturer's protocol (Sigma‐Aldrich). Total RNA of three biological replicates from PC3MPRO4 control cells and two biological replicates of PC3MPRO4 shDMTF1β_1 and shDMTF1β_2 were sent to Microsynth (Switzerland) for library preparation and mRNA sequencing (RNA‐seq). The generated reads were mapped to the human reference genome (GRCh38/hg38). The level of gene expression was assessed after RPKM normalization and log_2_ transformation. Genes with mean values of log_2_ RPKM > 3 for at least one of the conditions were kept in the analysis. The data set was analyzed by two‐way ANOVA. After statistical analysis, genes with significant expression differences (FDR‐*P* < 0.05) and fold differences of at least 1.5 were selected. PCA was used to map the variations between profiled samples. Data were clustered using Euclidian's method based on average linkage, and heatmaps were created using the normal distribution of the values.

### 2.14 Flag immunoprecipitation and mass spectrometry

HEK293T cells were transiently transfected with the desired plasmid via calcium‐phosphate precipitation. The following plasmids were used: pLV‐EF1α‐IRES‐Hyg, pLV‐EF1α‐Flag‐DMTF1β‐IRES‐Hyg, pLV‐EF1α‐Flag‐DMTF1ΔMHR‐IRES‐Hyg. After 48 h, cells were harvested, and Flag immunoprecipitation was performed using Flag magnetic beads (M8823; Sigma‐Aldrich/Merck) according to the manufacturer's protocol. The elution step was performed using 3xFlag peptide (F4799; Sigma‐Aldrich/Merck).

Eluted proteins were processed using the FASP protocol [23]. Briefly, proteins were reduced with 1 mm DTT and alkylated with 5.5 mm IAA on a 10 kDa molecular weight cut‐off filter (horizontal Vivacon 500, Sartorius) prior to trypsin digestion ON at 37 °C (trypsin : protein ratio of 1 : 100). Resulting peptides were desalted using STAGE tips as described [24] and subjected to LC–MS/MS analysis on a QExactive HF‐X mass spectrometer coupled to an EasyLC 1000 nanoflow‐HPLC. The mass spectrometer was operated in the data‐dependent mode; after each MS scan (mass range m/z = 370–1750; resolution: 60 000) a maximum of twelve MS/MS scans were performed using a normalized collision energy of 28%, a target value of 10 000 and a resolution of 60 000. The MS raw files were analyzed using maxquant Software version 1.6.2.10 [25] for peak detection, quantification and peptide identification using a full‐length UniProt human database (March, 2016) and common contaminants such as keratins and enzymes used for digestion as reference. Carbamidomethyl cysteine was set as fixed modification, and protein amino‐terminal acetylation and oxidation of methionine were set as variable modifications. The MS/MS tolerance was set to 20 ppm and three missed cleavages were allowed using trypsin/P as enzyme specificity. Peptide and protein FDR based on a forwards‐reverse database were set to 0.01, minimum peptide length was set to 7, and minimum number of peptides for identification of proteins was set to one, which must be unique. The “match‐between‐run” option was used with a time window of 0.7 min.

### 2.15 Gene ontology analysis

For gene ontology (GO) enrichment, the list of differently expressed genes/proteins was grouped into functional hierarchies. Enrichment scores were calculated using a chi‐square test comparing the proportion of the gene list in a group to the proportion of the background genes/proteins using GO resource database (geneontology.org), KEGG mapping (www.genome.jp/kegg), and Enrichr database (amp.pharm.mssm.edu/Enrichr). A score of 3 or higher corresponded to a significant GO enrichment (*P* < 0.05).

### 2.16 Gene set enrichment analysis

Gene set enrichment analysis (GSEA) was performed using GSEA software v.4 (Broadinstitute, Cambridge, UK). All pathway‐related genes were analyzed for enrichment using the Pathcards database (pathcards.genecards.org), GO datasets (geneontology.org), and the Broad Institute's Molecular Signatures Database (MSigDB).

### 2.17 Statistical analysis

When not differently indicated, experiments were performed in technical duplicates and biological triplicates. The GraphPad Prism software was used to analyze the generated data and to generate figures and diagrams. When not differently indicated, the nonparametric Mann–Whitney U‐test was applied to calculate significance values. Only *P*‐values below 0.05 were considered statistically significant.

## 3 Results

### 3.1 DMTF1β expression levels are positively associated with increased tumorigenic properties in breast and prostate cancer cells

DMTF1β was previously associated with breast cancer and mammary carcinogenesis [10, 13]. Breast cancer exhibits significant heterogeneity, distinguished by varying clinical behaviors and diverse histopathological and molecular characteristics. Key factors influencing treatment strategies include the expression levels of estrogen receptor (ER), progesterone receptor (PR), and human epidermal growth factor receptor 2 (HER2) [26]. Advances in gene expression analysis have further classified breast cancer into subtypes based on mRNA profiles: Luminal A, luminal B, HER2‐enriched and triple‐negative breast cancer (TNBC), each with distinct clinical features [27]. Among these, TNBCs have a poor prognosis, a higher likelihood of recurrence and metastasis, and limiting therapeutic options [28]. To further define the role of DMTF1β in breast cancer, we assessed endogenous DMTF1β mRNA and protein levels in a series of breast cancer cell lines representing different breast cancer subtypes. Firstly, we developed a DMTF1β‐specific qPCR (Fig. 1A). Specificity and efficiency of the DMTF1 splice variant‐specific TaqMan assays was determined using serial dilutions of DMTF1α and β expressing plasmids (Fig. S1A). We found variable levels of DMTF1β splice variant mRNAs in the breast cancer cells investigated (Fig. 1B). Consistent with this variability, protein expression is heterogeneous among the breast cancer cell lines. Notably, all three triple‐negative cell lines belonging to the mesenchymal subtype display protein expression levels exceeding the median value of the analyzed panel (Fig. 1C,D). To determine if DMTF1β expression correlates with increased mesenchymal and tumor initiating traits, we next assessed the expression of the widely used stem and mesenchymal markers CD44 and CD24 by flow cytometry [29]. Indeed, we found a significant, positive correlation (*r* ^2^ = 0.52, *P* < 0.01) between DMTF1β protein expression and a mesenchymal phenotype (% of CD44^+^/CD24^−^) in breast cancer cells (Fig. 1E).

**Fig. 1:**
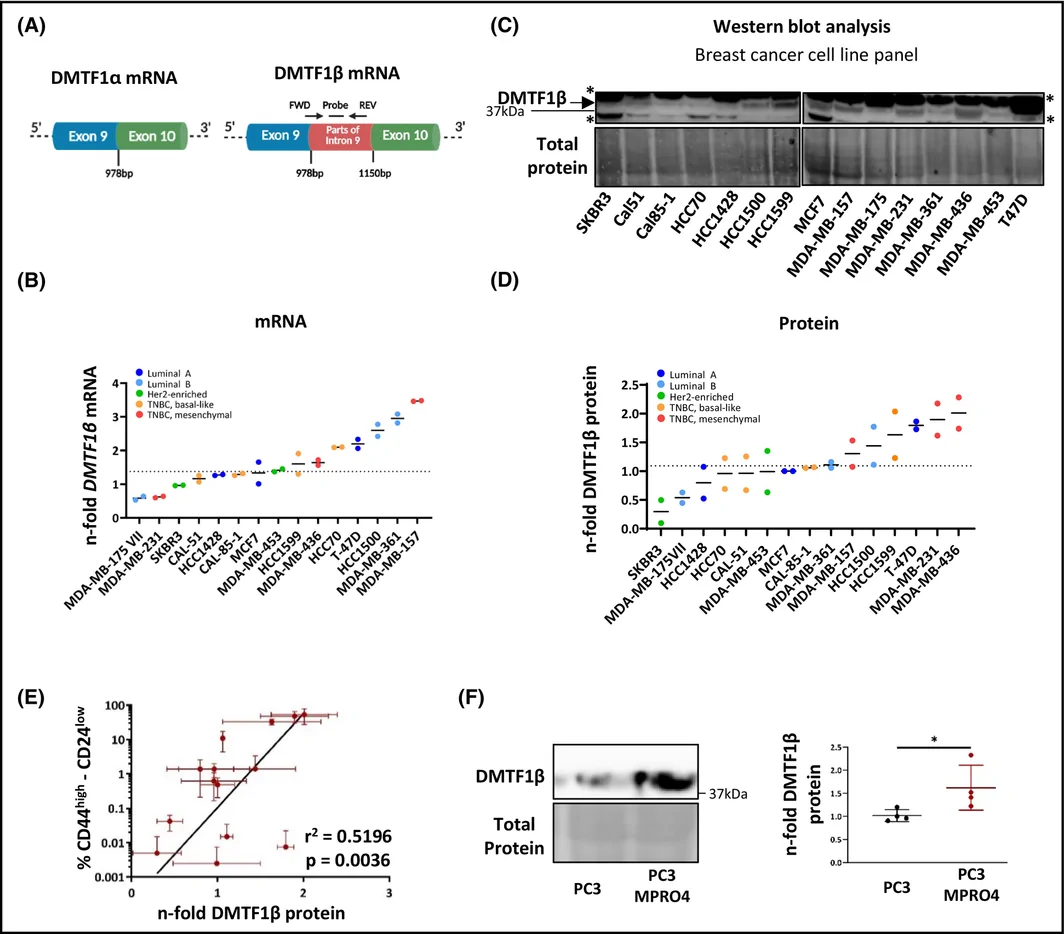
Increased DMTF1β expression is associated with a more aggressive cancer cell phenotype. (A) schematic representation of DMTF1 mRNA at the exon 9 and 10 alternative splicing site. The location of the designed primers and probes for DMTF1β‐specific qPCR is shown. For the DMTF1β specific qPCR product, the forward primer spans the Exon9‐Intron9 junction. Arrows and lines represent primers and probes, respectively. Created with BioRender.com. (B) DMTF1β mRNA levels in various breast cancer cells as determined by RT‐qPCR. Values were normalized to the housekeeping gene *Hydroxymethylbilane Synthase* (*HMBS*) and are shown as n‐fold regulation compared to the levels seen in MCF7 cells. Values shown are the mean expression across two independent experiments; within each experiment, data points represent the average of technical replicates. The median expression across cell lines is shown as a dotted line. (C) DMTF1β protein levels across the indicated breast cancer cell lines are shown. Asterisks mark nonspecific bands, while arrows indicate the DMTF1β band. (D) Quantification of DMTF1β protein levels of two independent experiments for which a representative western blot is shown in C. Levels are normalized to total protein and to MCF7. The plotted values represent the mean expression from two independent experiments. Each data point corresponds to the average of technical replicates within a single experiment. Dotted line indicates the median expression level of all cell lines. (E) Correlation between DMTF1β cellular protein levels (1C) and percentage of CD44^high^‐CD24^low^ cell surface expression within the cell line population as determined by flow cytometry is shown. The coefficient of determination (*r* ^2^) was calculated using Prism GraphPad software (Mann–Whitney U‐test (MWU), *P* = 0.0036). Error bars represent the standard deviation of three independent experiments; each performed in duplicate. (F) DMTF1β‐specific western blot of parental PC3 prostate cancer cells and a metastatic subline, PC3MPRO4. DMTF1β protein levels were quantified using imagej software and normalized to total protein and shown as n‐fold of PC‐3 cells. The graph shows average expression with standard deviation from three independent experiments. Statistical analysis was performed using GraphPad Prism (Mann–Whitney U‐test; *P ≤ 0.05).

Importantly, this finding is not restricted to breast cancer cells given that we detected significantly increased DMTF1β protein levels in the highly metastatic prostate cancer cell subline PC3MPRO4, when compared to the PC3 parental cells, which have a lower metastatic potential (Fig. 1F).

### 3.2 Depleting DMTF1β reduces breast and prostate cancer cell migration and invasion *in vitro* and *in vivo*

The process of migration and invasion that eventually leads to metastatic lesions is one of the hallmarks of cancer as described by Hanahan and Weinberg [30]. Given our previous findings showing a higher expression of DMTF1β in cells with increased metastatic activity, we aimed at testing whether alteration of the oncogenic DMTF1β isoform levels has an impact on migration and invasion. First, we knocked out the DMTF1 gene using CRISPR‐Cas9 technology. MCF7 cells were chosen as a well‐characterized, p53 wild‐type, luminal breast cancer model. In two MCF7 DMTF1 knockout cell clones, we re‐expressed the DMTF1β isoform to study specific and therefore DMTF1α‐independent effects (Fig. 2A). We did not observe any significant differences in cell proliferation upon DMTF1 depletion (Fig. S2A). However, knocking out all DMTF1 splice variants in MCF7 cells significantly decreased wound closure over time, while ectopic re‐expression of DMTF1β increased wound closure in both knockout cell clones (Fig. 2B). MCF‐7 cells with DMTF1 knocked out and ectopic expression of high levels of DMTF1β showed increased migration across a polycarbonate membrane (trans‐well migration) as compared to DMTF1 knockout cells (Fig. 2C). Furthermore, specifically knocking down DMTF1β in the metastatic MDA‐MB‐231 breast and PC3MPRO4 prostate cancer cell lines significantly reduced wound closure over time compared to control cells, without significantly affecting cell proliferation (Fig. 2D,E, Fig. S2B,C). We further performed trans‐well invasion assays that involve invasion across Matrigel, mimicking migration through basement membrane. Silencing DMTF1β in MDA‐MB‐231 and PC3MPRO4 cells resulted in significantly decreased trans‐well invasion compared to vector controls (Fig. 2F). In summary, high levels of DMTF1β expression in breast and prostate cancer cells are associated with more migration and invasion. Since migration and invasion are linked to a more mesenchymal phenotype [31], we measured the epithelial marker *CDH1* (E‐Cadherin) in our cell line models. We found that DMTF1β depletion significantly increased *CDH1* mRNA in MDA‐MB‐231 and PC3MPRO4 cells. In contrast, overexpression of DMTF1β in MCF‐7 DMTF1 knockout cells decreased *CDH1* mRNA levels (Fig. S2D).

**Fig. 2:**
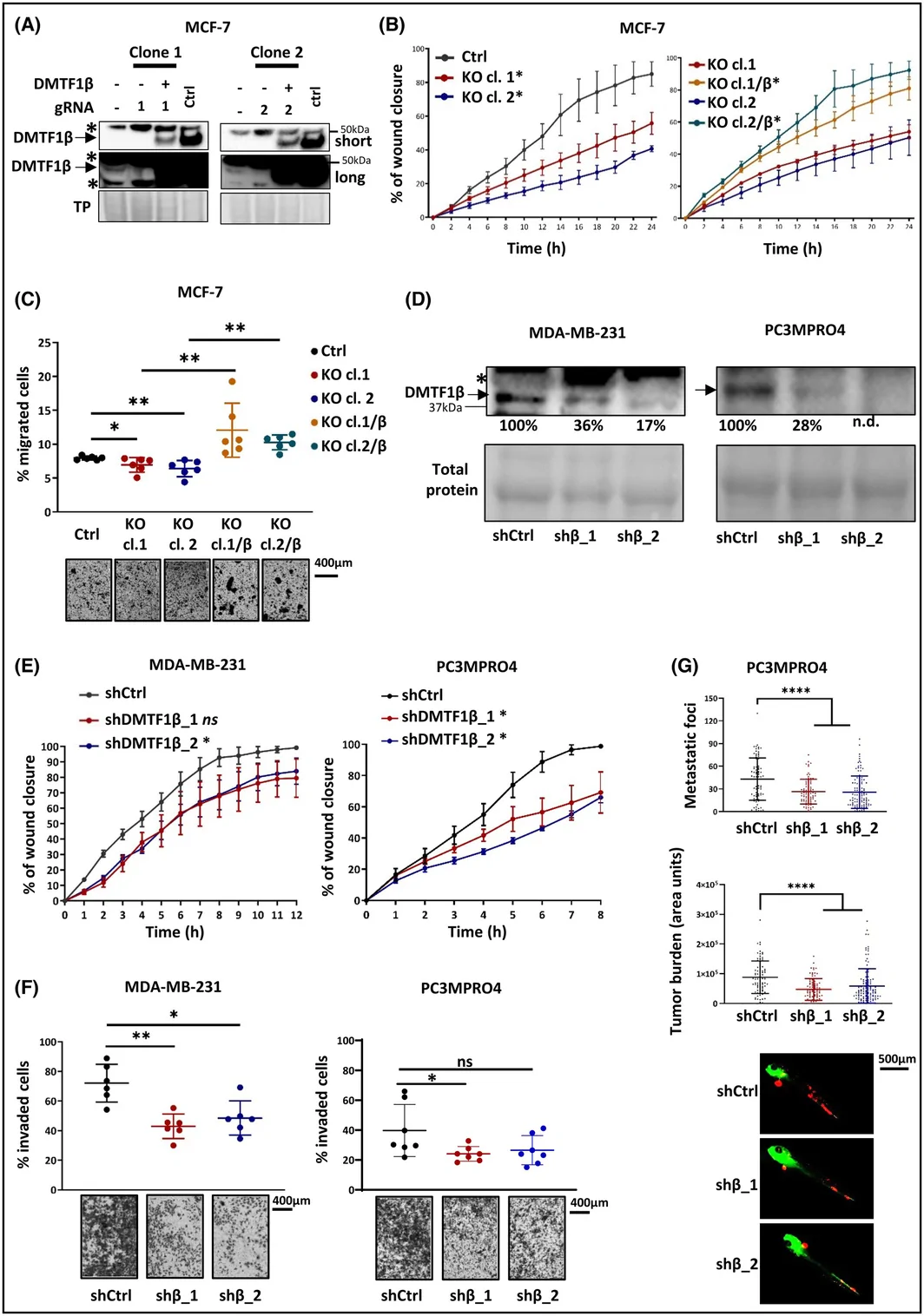
DMTF1β supports cancer cell migration and invasion. (A) Validation of MCF7 DMTF1 knockout clones by western blotting. Ectopic DMTF1β expression in MCF7 cells served as a positive control (ctrl). Short and long exposures are shown. Total protein (TP) served as loading control. Asterisks indicate nonspecific bands, while arrows denote the DMTF1β band. (B) Cell migration was assessed via wound closure assay using Cell‐IQ live cell imaging. Images were taken every hour and wound closure was measured by Activision software. Error bars represent the standard deviation from three independent experiments with two technical replicates each. Two‐way ANOVA (TWA) test was used to test significance between groups (*P ≤ 0.05). (C) Trans‐well migration assay of MCF‐7 DMTF1 knockout clones (KO cl.1 and cl.2) with and without rescue for DMTF1β (β). Migrated cells were PFA‐fixed and stained with crystal violet. For each well the average of five pictures was calculated. Pictures were quantified using imagej software. Scale bar corresponds to 400 μm. Error bars represent the standard deviation of three independent experiments; each performed in duplicate. MWU was applied to assess statistical differences (*P ≤ 0.05, **P < 0.01). (D) Representative Western Blot of DMTF1β protein levels in MDA‐MB‐231 and PC3MPRO4 cells under control conditions (shCtrl) and following DMTF1β knockdown (shβ_1, shβ_2). Quantification was performed using imagej and normalized to total protein levels as well as to the corresponding shCtrl condition for each cell line. Nonspecific bands are marked with asterisks, and the DMTF1β band is indicated by arrows. (E) Migration of control (shCtrl) or DMTF1β knockdown cells was assessed as in 2B. Graph shows mean with standard deviation of three independent experiments with two technical replicates each. TWA test was used to show significance between groups (*P ≤ 0.05). (F) Invasion was assessed by trans‐well invasion assays using Matrigel to coat the transwell membrane. Invaded control (shCtrl) or two different DMTF1β knockdown lines (shDMTF1β_1 and shDMTF1β_2) were PFA‐fixed and stained with crystal violet. Each data point represents the average of five pictures from one well. The data derive from three independent, each with two or three technical replicates. Pictures were quantified using imagej software. Scale bar corresponds to 400 μm. Differences between groups was assessed by MWU (*P ≤ 0.05, **P < 0.01). (G) Zebrafish xenografts were performed using mCherry labeled PC3MPRO4 cells that were implanted in two GFP‐positive zebrafish embryos. Fluorescent image acquisition was performed using a Leica MZ16FA stereo microscope. Tumor burden was assessed measuring the intensity of mCherry, while metastatic foci were assessed counting mCherry dots. Scale bar corresponds to 400 μm. Results shown means ± SD (MWU, ****P < 0.0001).

To determine the metastatic potential of our DMTF1 modified prostate cancer cell line model *in vivo*, we performed zebrafish xenograft experiments. When injected in the duct of Cuvier of zebrafish embryos, DMTF1β knockdown PC3MPRO4 cells showed significantly reduced total tumor burden and metastatic foci as compared to cells with the vector only (Fig. 2G). These findings clearly suggest that DMTF1β expression in breast and prostate cancer cells supports migration and invasion.

### 3.3 Transcriptomic analysis of PC3MPRO4 DMTF1β knockdown prostate cancer cells revealed a reduction of autophagy‐related pathways

To molecularly decipher how DMTF1β might contribute to increased cancer cell motility, we performed RNA‐seq analysis of PC3MPRO4 DMTF1β knockdown and control cells. The PC3MPRO4 subline was chosen as it expresses high levels of DMTF1β as compared to the PC3 parental cell line (Fig. 1F). The principal component analysis (PCA) of 3 biological replicates of control cells and two biological replicates of two cell lines each harboring a different shRNA targeting *DMTF1β* is shown in Fig. 3A. With a threshold set at a 1.5‐fold change, we identified 868 significantly downregulated and 189 upregulated genes in cells expressing shDMTF1β_1, as compared to 480 significantly downregulated and 88 upregulated genes in cells expressing shDMTF1β_2, relative to control cells (Fig. 3B; Table S1). Next, we assessed the genes that were differently expressed in both knockdown cell lines compared to control. We found 285 differentially expressed genes common in the shDMTF1β_1 and _2 sample (Fig. 3C). 255 of these genes were down‐ and 30 upregulated. In addition to the identified migration‐related genes, many autophagy‐related genes were downregulated in the DMTF1β knockdown cells as assessed by gene ontology (GO) and gene set enrichment analysis (GSEA) (NES = −1.19) (Fig. 3D,E). We validated the downregulation of the key autophagy genes *WIPI1* and *VPS34* in DMTF1β depleted cells by qPCR. ATG mRNA levels of both genes were significantly downregulated in PC3MPRO4 cells expressing shRNAs targeting *DMTF1β* (Fig. 3F,G, right panels), whereas only WIPI1, but not VPS34, was lower on protein level upon DMTF1β knockdown (Fig. S3A,B). VPS34 mRNA expression was also significantly downregulated in MDA‐MB‐231 cells transduced with shRNAs targeting *DMTF1β*, whereas *WIPI1* mRNA only shows a trend towards lower expression in these cells (Fig. 3F,G, middle panels). Re‐expression of DMTF1β in PC3MPRO4 DMTF1β knockdown cells partially, although not significantly, rescued the WIPI1 protein levels. Interestingly, removing the β‐specific domain did not rescue the low WIPI1 protein levels in PC3MPRO4 DMTF1β knockdown cells (Fig. S3A,B). Similarly, DMTF1β re‐expression in MCF7 DMTF1 knockout cells resulted in a significant rescue of *VPS34* and *WIPI1* mRNA levels (Fig. 3F,G, left panels). Taken together, our findings revealed that DMTF1β is associated with migration, invasion, and, in addition, autophagy pathways.

**Fig. 3:**
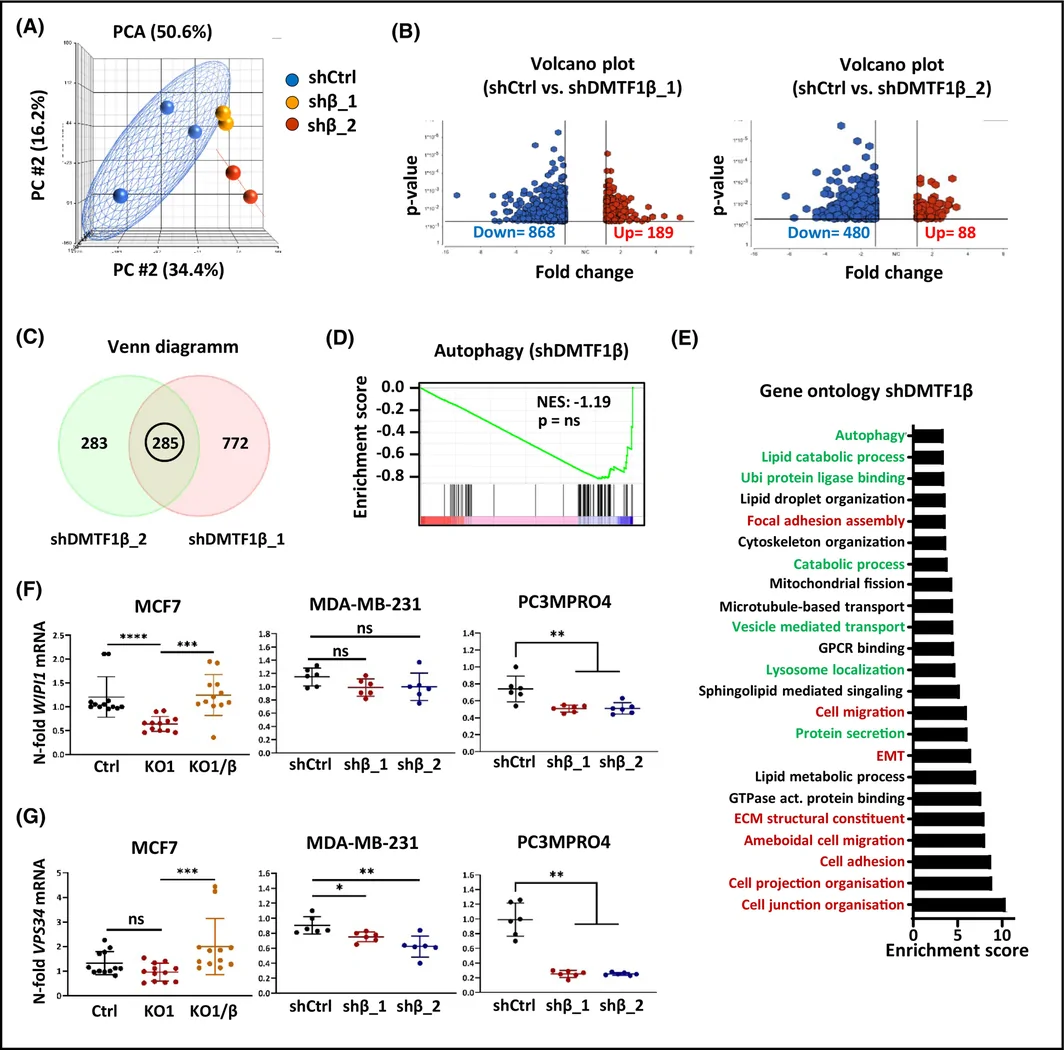
Transcriptomic analysis links DMTF1β knockdown to autophagy and cell migratory pathways. (A) Principal component analysis (PCA) of DMTF1β silenced (shβ_1 and shβ_2) PC3MPRO4 cells using two independent shRNAs (*n* = 2; per each shRNA) and control cells (shCtrl, *n* = 3). (B) Volcano plots representing differentially expressed genes in shDMTF1β_1 or shDMTF1β_2 versus controls. Red and blue dots mark genes with significantly increased or decreased expression, respectively (Fold change ≥1.5 and *P*‐value <0.05). (C) Venn diagram comparison of differentially expressed genes using two different shRNAs (shDMTF1β_1 or shDMTF1β_2 vs. control). (D) Gene set enrichment analysis (GSEA) representing the normalized enrichment score (NES) of the autophagy gene set (shDMTF1β vs. control). GSEA was performed using a signal‐to‐noise ratio weighted by gene‐level scores. (E) Gene ontology (GO) analysis of downregulated intersection genes (shDMTF1β vs. control). A score of ≥3 indicates significant GO enrichment. Green and red represent autophagy‐ and migration‐related pathways, respectively. (F, G) *WIPI1* and *VPS34* mRNA levels were quantified by RT‐qPCR. mRNA levels were normalized to the housekeeping gene *HMBS* and the control (Ctrl) according to the ΔΔCt method. Statistical differences were calculated using Prism GraphPad software. Data shown in mean ± SD of 3 or 4 independent experiments in duplicates (MWU, ns, >0.05, *P ≤ 0.05, **P < 0.01, ***P < 0.001, ****P < 0.0001).

### 3.4 DMTF1β supports autophagic activity in breast and prostate cancer cells

Autophagy is a key catabolic process responsible for the degradation and clearance of intracellular entities such as organelles and proteins [18]. Since autophagy can affect cancer cell migration and metastasis in a context‐dependent manner [32, 33, 34] and since our pathway analysis above shows that DMTF1β is associated with autophagy, we hypothesized that DMTF1β increases autophagic activity. We assessed autophagic activity in our cell panel, also referred to as autophagic flux, by quantifying the accumulation of lipidated LC3B (LC3B‐II) protein on western blot and LC3B puncta formation by immunofluorescence while blocking autophagy using Bafilomycin A1. Knocking out DMTF1 in MCF7 cells or specifically knocking down DMTF1β in MDA‐MB‐231 and PC3MPRO4 cancer cells significantly decreased LC3B‐II protein accumulation and LC3B dot formation in the presence of Bafilomycin A1 as compared to control cells. Re‐expressing DMTF1β in MCF7 DMTF1 knockout cells demonstrated significantly increased autophagic flux (Fig. 4A,B). Next, we performed long‐lived protein degradation assays (LLPDA) to exclude any LC3B function in autophagy‐independent processes [35]. Bafilomycin A1‐dependent (= lysosomal) proteolysis was significantly reduced in MCF7 DMTF1 knockout cells as well as in MDA‐MB‐231 and PC3MPRO4 DMTF1β knockdown cells when compared to vector only controls (Fig. 4C). Similar results were obtained when measuring VPS34‐dependent proteolysis (Fig. 4D), which more specifically assesses macroautophagy.

**Fig. 4:**
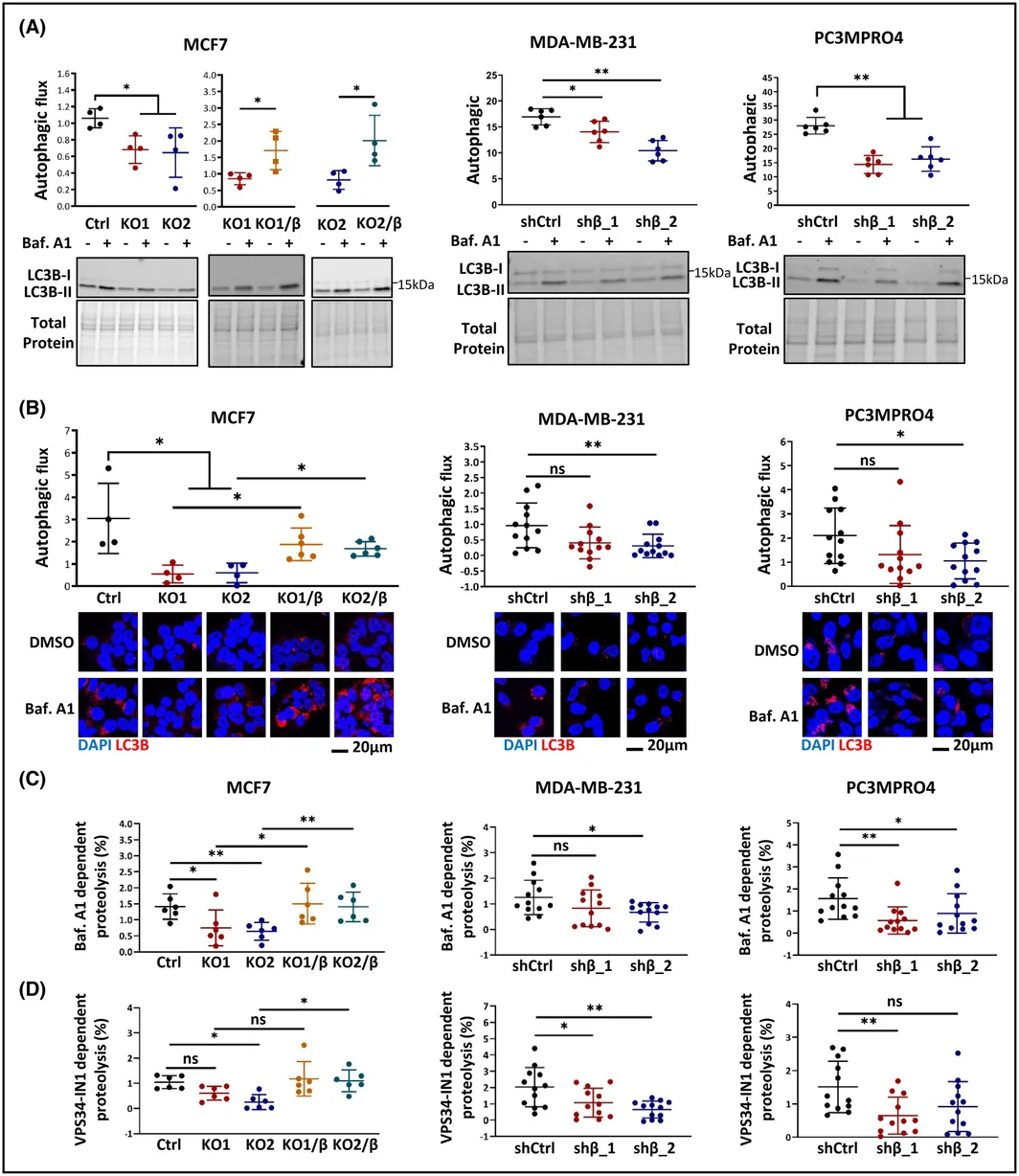
DMTF1β is an autophagy activator. (A) Autophagy flux was measured in MCF‐7 knockout of DMTF1 clones (KO1 and KO2) ± DMTF1β rescue (β) as well as in MDA‐MB‐231 and PC3MPRO4 DMTF1β knockdown (shβ_1 and shβ_2) and control (shCtrl) cells. Cells were treated with Bafilomycin A1 (Baf. A1). Western blotting for LC3B‐I and II is shown. Protein levels were normalized to total protein before calculating the autophagic flux by subtracting the lipidated LC3B (LC3B‐II) levels in DMSO‐treated cells from the amount of lipidated LC3B (LC3B‐II) in Bafilomycin A1 (Baf.A1) treated samples. Quantification was performed using imagej software. Graphs represent mean ± SD from 4 independent experiments. (MWU *P ≤ 0.05, **P < 0.01). (B) Immunofluorescence pictures of LC3B and quantification are shown. Dots were calculated using imagej software. Scale bar indicates 20 μm. Autophagic flux was assessed by subtracting the amount of LC3B dots in DMSO‐treated cells from the number of puncta in the respective Baf.A1‐treated condition. Each data point represents the mean flux of 6 pictures from one independent experiment. Error bars represent the SD between the four independent experiments calculated using GraphPad Prism (MWU, *P ≤ 0.05, **P < 0.01). Blue = nucleus (DAPI), red = LC3B (Cy3). (C, D) Cells were labeled with C^14^‐valine before the long‐lived protein degradation assay (LLPDA) was performed. Radioactivity was measured by liquid scintillation counting using a Microbeta2 instrument. Baf. A1 or VPS34‐IN1‐dependent proteolysis was calculated by subtracting the amount of proteolysis in presence of Baf. A1 and VPS34‐IN1 from the amount degraded in absence of autophagy inhibitors, respectively. Statistical analyses were performed using Prism GraphPad software. Data are shown in mean ± SD from three independent experiments with two technical replicates each (MWU, ns >0.05, *P ≤ 0.05, **P < 0.01).

Lastly, we confirmed our observations that DMTF1β stimulates autophagy using the HyD‐LIR(TP)‐GFP sensor [36]. This construct allows the detection of ATG8 positive structures in a LIR (LC3‐interacting)‐motif‐dependent manner. It is worth mentioning that it contains the LIR domain of TP53INP2, which preferentially interacts with the ATG8‐family members GABARAP and GABARAPL1 [36] and can therefore be regarded as another LC3B independent assay. Using this assay, we found that PC3MPRO4 and MDA‐MB‐231 sh*DMTF1β* cells displayed reduced basal autophagic flux when compared to control cells (Fig. S4A).

Given that DMTF1α can contribute to p53 protein stabilization via p14^ARF^ activation and DMTF1β can attenuate DMTF1α functions, we next tested if the DMTF1β‐mediated migration and invasion is p53‐dependent. The fact that MDA‐MB‐231 cells express mutant p53 (p53^R280K^) and PC3MPRO4 cells are p53 null [37, 38], and both show DMTF1β‐dependent basal autophagy hints at a p53‐independent function of DMTF1β in autophagy regulation. To further examine this possibility, we silenced wild‐type p53 using two different siRNAs in MCF7 breast cancer cells. In MCF7 control and DMTF1 knockout cells and knockout cells re‐expressing DMTF1β, knocking down p53 did not significantly alter autophagic flux nor cell migration (Fig. S4B,C).

Together, these findings demonstrate that DMTF1β induces autophagic flux in breast and prostate cancer cells, as assessed by multiple autophagy assays. This autophagy modulating function of the β isoform is independent of full‐length DMTF1α as well as p53.

### 3.5 DMTF1β protein interacts with ATG proteins via its β‐specific domain

To identify specific cellular binding partners of DMTF1β, we performed mass spectrometry of immunoprecipitated, ectopically expressed, and Flag‐tagged DMTF1β and DMTF1∆MHR proteins and their binding partners. DMTF1∆MHR is identical to DMTF1β but lacks the β‐specific amino acids (Fig. 5A). Data obtained from 3 biological replicates of ectopically expressed and immunoprecipitated Flag‐control, Flag‐DMTF1β, and Flag‐DMTF1∆MHR samples (PCA = 71%) revealed 563 differently binding proteins (1.3‐fold, *P* < 0.05, neg. values filtered) when comparing Flag‐control to Flag‐DMTF1β immunoprecipitants, while 353 proteins (1.3‐fold, *P* < 0.05, neg. values filtered) bound differently to the Flag‐DMTF1∆MHR when compared to Flag‐control samples. We observed that 240 proteins were enriched in the Flag‐DMTF1β and Flag‐DMTF1∆MHR immunoprecipitants compared to the control. These proteins represent binding partners of the shared protein region of both constructs. Furthermore, we found 323 proteins that were co‐immunoprecipitated with DMTF1β, but not Flag‐DMTF1∆MHR, which were also enriched compared to control. These proteins may represent specific binding partners of the β‐specific domain (Fig. 5B). Of note, proteins associated with early autophagy such as ULK1, ATG13, and WD Repeat Domain 45B (WDR45B) and with mitophagy including Prohibitin2 (PHB2) and translocase of outer mitochondrial membrane (TOMM) family members were specifically precipitated by DMTF1β (Fig. 5C). Gene ontology as well as gene enrichment analysis revealed that only β‐specific domain binding partners, but not those of DMTF1∆MHR lacking the β‐specific domain, were significantly associated with the autophagy pathway (Fig. 5D,E). In conclusion, the DMTF1β specific protein interactome suggests a direct involvement of DMTF1β in autophagy.

**Fig. 5:**
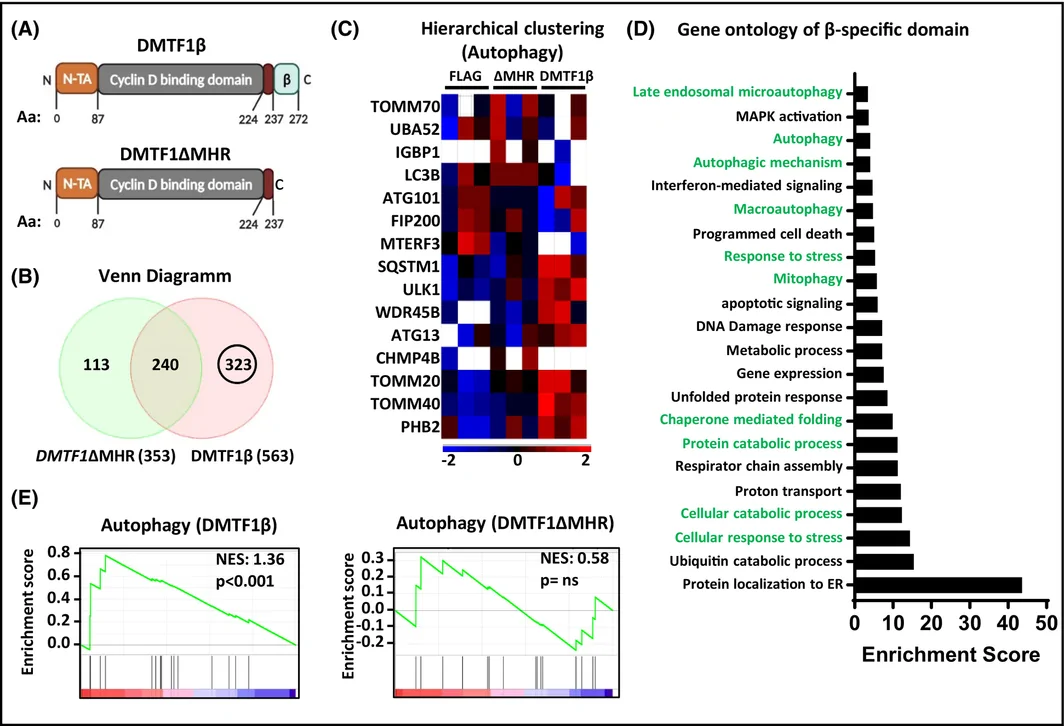
The DMTF1β‐specific domain interacts with autophagy‐related proteins. (A) Schematic illustration of human DMTF1β with previously identified domains as well as a deletion mutant lacking the β‐specific domain (DMTF1ΔMHR). (B) Venn diagram showing the intersection of 293 T cellular proteins interacting with ectopically expressed DMTF1β, DMTF1ΔMHR, or FLAG (control) proteins. See Methods for experimental procedures of cellular IP of DMTF1β, DMTF1ΔMHR, or FLAG (control) proteins and mass spectrometry identification of isolated HEK293T cellular proteins. (C) Heat map of autophagy proteins, which are present in DMTF1β, DMTF1ΔMHR, and FLAG isolated HEK293T cellular proteins (n = 3 biological replicates). Blue and red represent low and high abundance, respectively. (D) Gene ontology (GO) analysis of positively enriched proteins of the β‐specific domain (DMTF1β vs. DMTF1ΔMHR). A score of ≥3 indicates significant GO enrichment. (E) Gene set enrichment analysis (GSEA) representing the normalized enrichment score (NES) of autophagy related proteins (DMTF1β vs. FLAG and DMTF1ΔMHR vs. FLAG). Blue and red represent low and high abundance, respectively (*P* < 0.001). GO analysis was performed using Fisher's exact test. A GO enrichment score ≥3 (−Log_10_ *P*‐value) indicates significant enrichment.

### 3.6 DMTF1β binds to ULK1 protein and supports its stability

To confirm that DMTF1β interacts with components of the ULK1 complex, Flag‐DMTF1β and HA‐ULK1 or Flag‐GFP and HA‐ULK1 expression plasmids were transiently transfected in HEK293T cells, and a Flag immunoprecipitation (IP) was performed to isolate binding partners. We found that DMTF1β interacts with ULK1 and ATG13 (Fig. 6A, Fig. S5A). Importantly, Flag‐DMTFβ also interacts with endogenous ULK1 and ATG13, but not with Beclin‐1 nor VPS34 (Fig. 6B, Fig. S5B). To test if the binding interactions are specific to the β‐specific domain, an IP was performed using the Flag‐DMTF1ΔMHR construct, which lacks the β‐specific domain. As shown in Fig. 6B (and Fig. S5B), Flag‐DMTF1ΔMHR binds endogenous ULK1 and ATG13, but with 50% less protein as compared to Flag‐DMTF1β (Fig. S5C). To further validate our Co‐IP results, we performed *in situ* proximity ligation assay (PLA) with DMTF1β knockdown PC3MPRO4 cells rescued with DMTF1β‐HiBiT. Red fluorescent dots, indicative for close proximity of two proteins, were detected for DMTF1β‐HiBiT and ULK1. Importantly, the number of proximity spots were significantly lower in DMTF1β‐HiBiT‐negative cells (Fig. 6C). To exclude overexpression artifacts, we used CRISPR/Cas9‐mediated genome editing to knock in a HiBiT tag at the C terminus of the endogenous DMTF1β gene. HiBiT is part of a split‐luciferase system, in which the abundance of HiBiT‐fusion proteins is reflected by the bioluminescence signal intensity following cell lysis and addition of LgBiT (Fig. S5D). After generating single‐cell clones and measuring luminescence, we identified an MCF‐7 cell clone expressing endogenously HiBiT‐tagged DMTF1β (Fig. S5E). To exclude the possibility that the signal resulted from off‐target integration, we specifically knocked down DMTF1β in these cells using shRNA delivered by lentiviral vectors. DMTF1β knockdown led to a marked reduction in luminescence, as measured by the HiBiT lytic assay (Fig. S5F), indicating that the detected signal originates from HiBiT‐tagged DMTF1β. Together, these data confirm successful endogenous tagging of the DMTF1β splice variant. Next, we further determined proximity of ULK1 and DMTF1β in MCF‐7 cells with endogenous HiBiT‐tagged DMTF1β. In agreement with the very low expression of DMTF1β, we found very few, but significantly more ULK1‐DMTF1β‐HiBiT proximity spots in HiBiT‐tagged than in parental MCF‐7 cells. There was a trend to more interactions between ULK1 and DMTF1β upon Torin1‐treatment (Fig. S5G,H). Taken together, these observations suggest that DMTF1β interacts with components of the ULK1 complex. We next explored the consequences of DMTF1β–ULK1 complex interaction. Knocking down DMTF1β decreased the expression levels of ULK1, but not ATG13 (Fig. S5I,J). To ensure the specificity of DMTF1β‐mediated ULK1 protein expression, we performed a rescue experiment by introducing shRNA‐resistant forms of DMTF1β into PC3MPRO4 sh*DMTF1β* cells, which restored ULK1 expression levels (Fig. 6D). Notably, *ULK1* mRNA levels remained unchanged in DMTF1β‐depleted cells (Fig. S5K). We therefore investigated whether ULK1 reduction resulted from enhanced proteasomal degradation. As is shown in Fig. 6E, treatment with proteasome inhibitor MG132 rescued lower ULK1 levels in DMTF1β‐depleted cells. In agreement, cycloheximide chase assay revealed a significantly lower ULK1 protein stability upon knockdown of DMTF1β as compared to control cells. Importantly, ULK1 protein stability was rescued with DMTF1β, but not with the DMTF1ΔMHR construct that lacks the β‐specific domain (Fig. 6F,G). Similarly, migratory capacity across a membrane was significantly reduced in DMTF1β‐depleted cells and rescued upon DMTF1β overexpression. DMTF1ΔMHR did not significantly rescue the migratory phenotype of DMTF1β‐depleted cells (Fig. 6H). In summary, our data suggest that DMTF1β regulates ULK1 protein stability and cancer cell migration and that this phenotype is largely dependent on the β‐domain.

**Fig. 6:**
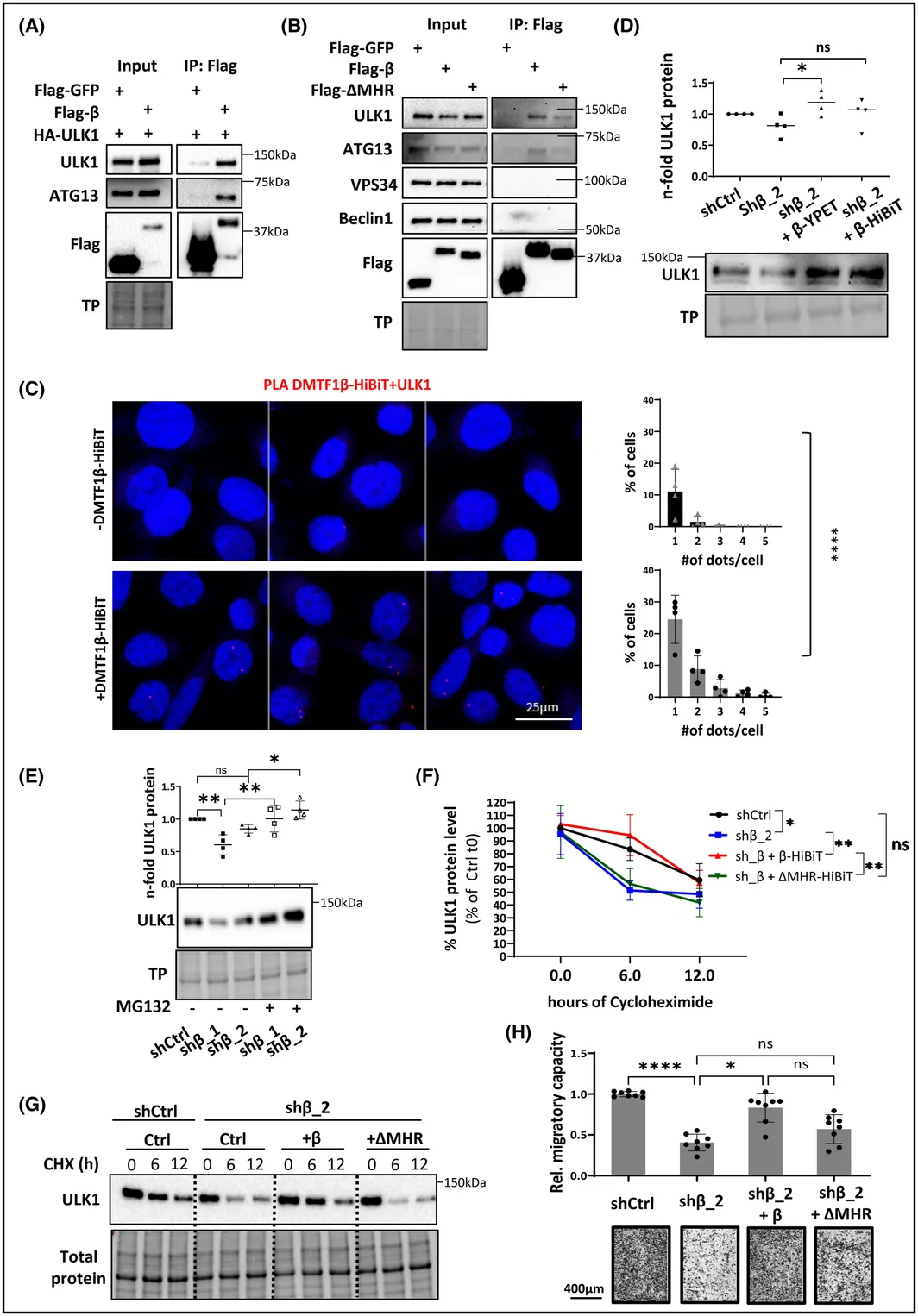
DMTF1β binds and modulates ULK1 protein levels. (A) HEK293T cells were co‐transfected with indicated plasmids for 48 h, then cell lysates were subjected to IP using anti‐Flag magnetic beads. Flag‐GFP, Flag‐DMTF1β, HA‐ULK1 and endogenous ATG13 in the immunoprecipitants were detected by western blotting. A repetition is shown in Fig. S5A. (B) HEK293T cells were transfected with Flag‐GFP, Flag‐DMTF1β or Flag‐DMTF1ΔMHR for 48 h. Cell lysates were subsequently subjected to IP using anti‐Flag magnetic beads. Endogenous ULK1, ATG13, VPS34 and Beclin‐1 proteins were detected in the IP sample by western blotting. The results of a second IP are shown in Fig. S5B. (C) PC3MPRO4 cells ectopically expressing a control vector (‐DMTF1β‐HiBiT) or a HiBiT‐tagged DMTF1β (+DMTF1β‐HiBiT) were subjected to the proximity ligation assay (PLA) with primary antibodies against ULK1 and HiBiT. Nuclei were stained with DAPI. The cells were imaged by confocal microscopy and shown are three representative fields. Quantification of the percentage of cells containing 1, 2, 3, 4 or 5 dots is shown on the right. Error bars represent SD of four independent experiments; each data point corresponds to one experiment, with percentages calculated from cells on six images each. Scale bar corresponds to 25 μm. Ordinary TWA was used to assess differences. ****P < 0.0001. (D) Western blot analysis and quantification of endogenous ULK1 protein in PC3MPRO4 control (shCtrl) or DMTF1β knockdown (shβ_2) cells ectopically expressing two differentially tagged shRNA‐resistant DMTF1β constructs. The graph shows mean ± SD of 4 independent experiments. One‐way ANOVA followed by Holm‐Šídák's multiple comparisons test was used to assess differences in selected groups (ns, not significant, *P ≤ 0.05, **P < 0.01). (E) Western blot analysis and quantification of endogenous ULK1 protein in cell lysates extracted from PC3MPRO4 control (shCtrl) or DMTF1β knockdown (shβ_1 or shβ_2) cells. Where indicated cells were treated with MG132 for 8 h ULK1 levels were quantified using ImageJ and normalized to total protein and shCtrl. Quantification and statistical analysis as in D. (F, G) ULK1 protein stability was assessed in PC3MPRO4 cell lines following treatment with Cycloheximide for 0, 6, and 12 h. Cells expressed either a control shRNA (shCtrl) or an shRNA targeting DMTF1β (shβ_2). Where indicated cells were additionally transfected with either a DMTF1β–HiBiT expression construct (β‐HiBiT) or a DMTF1β mutant lacking the β‐domain (DMTF1ΔMHR–HiBiT). Graph shows mean ± SD of 4 independent experiments. Selected groups were analyzed by two‐way ANOVA followed by Sidak's *post hoc* test (ns, not significant, *P ≤ 0.05, **P < 0.01). A representative western blot for ULK1 is shown in G. (H) Cell migration of PC3MPRO4 cells described in 6F‐G was assessed by trans‐well migration assays. Migrated cells were PFA‐fixed and stained with crystal violet. 4 pictures of each well were taken using the EVOS FL microscope. Pictures were quantified using imagej software, Graphs represent mean ± SD of four independent experiments with two replicates each. Statistical significance for selected groups was determined using two‐way ANOVA followed by Kruskal–Wallis test for multiple comparisons (ns, not significant, ****P ≤ 0.0001). Scale bar = 400 μm.

### 3.7 DMTF1β‐mediated cancer cell motility is driven by autophagy

There is evidence that autophagy can support or attenuate cancer cell migration depending on the context [32, 33, 34, 39]. Our findings above suggest that oncogenic DMTF1β is directly involved in autophagy activation and increased cell migration. Therefore, we speculated that DMTF1β mediated autophagy is responsible for the observed enhanced migration and invasion in cancer cells. First, we evaluated the optimal working concentration for the autophagy inhibitors VPS34IN1 (VPS34 inhibitor) and MRT68921 (ULK1 inhibitor) in MCF7 breast cancer cells following 24 h of treatment, which reflects the duration of the migration assay. Autophagic flux was assessed by mCherry‐GFP‐LC3B FACS analysis (Fig. S6A,B). Blocking autophagy using VPS34IN1 and MRT68921 autophagy inhibitors significantly reduced migration in DMTF1β expressing MCF7 cells, while the reduction of migration in the DMTF1‐knockout clones was moderate or absent as assessed by wound closure assays (Fig. 7A, Fig. S6C). To support our findings based on pharmacological autophagy inhibition, we knocked down VPS34 and ULK1 gene expression in MCF7 DMTF1 cell line models. ATG gene knockdown efficiency and autophagic flux were assessed by western blotting. Autophagic flux was reduced more than 50% across all the ATG gene knockdowns in all MCF7 cell models (Fig. S6D,E). In line with our findings using pharmacological inhibitors, knocking down VPS34 and ULK1 gene expression significantly decreased migration of DMTF1β expressing cells (Fig. 7B). Next, we aimed at increasing autophagic activity in DMTF1β silenced cells displaying low autophagic activity. We ectopically expressed the autophagy‐related gene Beclin1 in control and DMTF1β knockdown PC3MPRO4 cells to increase autophagic flux [40, 41]. Beclin1 overexpression and autophagic activity was assessed via western blotting. Ectopic Beclin1 expression rescued autophagic activity in the DMTF1β silenced PC3MPRO4 cells (Fig. 7C). Importantly, increasing autophagic activity in DMTF1β knockdown significantly rescued invasion as assessed by trans‐well invasion assays (Fig. 7D). Taken together, we found that inhibition of DMTF1β‐induced autophagy resulted in reduced migration of cancer cells, while ectopic expression of Beclin1 restored autophagy and invasion in PC3MPRO4 DMTF1β knockdown cells.

**Fig. 7:**
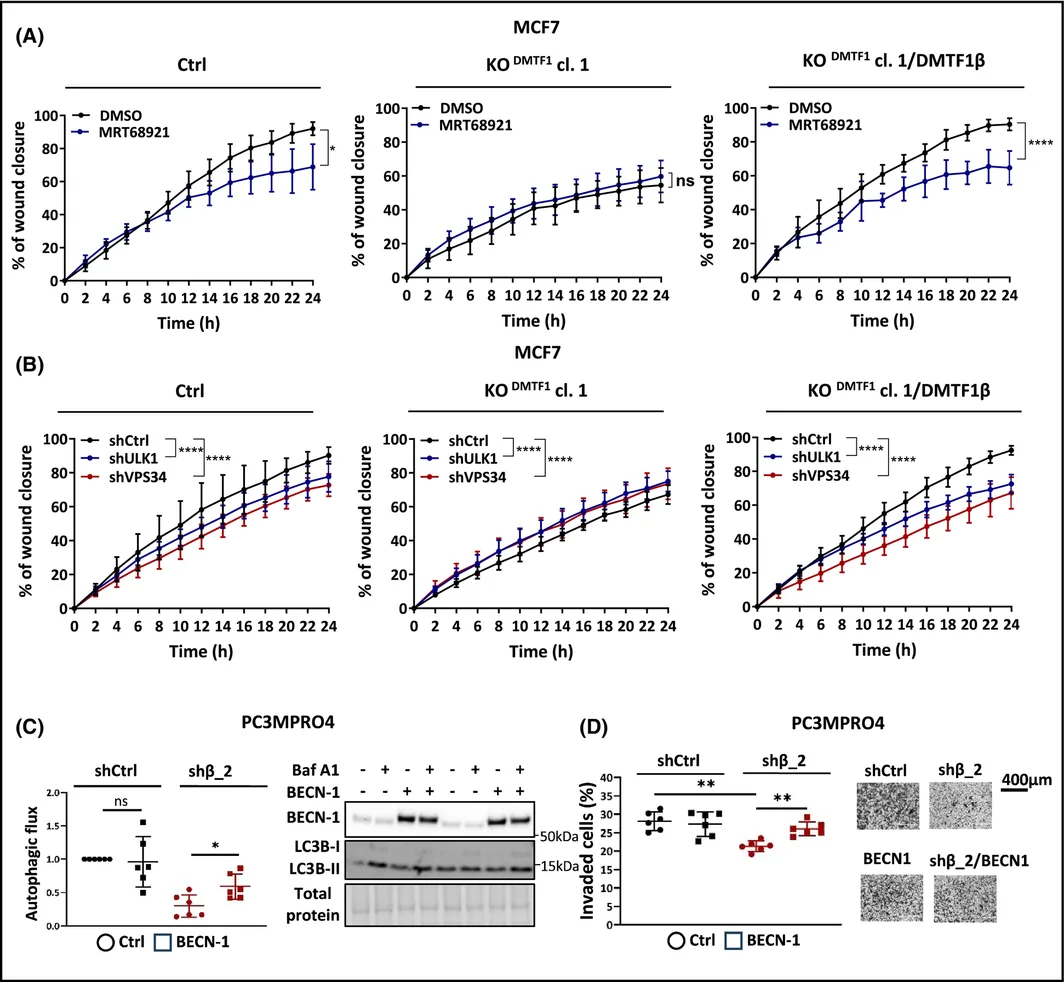
DMTF1β‐mediated autophagy drives cancer cell migration. (A, B) Migration was assessed by wound closure assay in MCF‐7 control (KO Ctrl) and DMTF1 knock‐out (KO^DMTF1^ cl.1) clones ± DMTF1β rescue (KO^DMTF1^ cl.1/DMTF1β) using Cell‐IQ live cell imaging. Autophagy was blocked using MRT68921 (A), or shRNAs targeting either *ULK1* (sh*ULK1*) or *VPS34* (sh*VPS34*) (B). Images were taken every hour of the same wound area and wound closure was measured by Activision software. Error bars show standard deviation from four independent experiments with two replicates each. TWA followed by Dunnett's multiple comparison test was used to assess significance between groups (ns, not significant, *P ≤ 0.05, ****P ≤ 0.0001). (C) LC3B flux (autophagic flux) was calculated in control (shCtrl) and shDMTF1β (β_2) PC3MPRO4 cells with and without ectopic expression of Beclin‐1 (BECN‐1) based on LC3B western blotting in presence or absence of Bafilomycin A1 (Baf A1). After normalization to total protein, autophagic flux was defined as LC3B‐II (Baf.A1) − LC3B‐II (DMSO). Bar graphs represent SD of 6 independent experiments. Significance was assessed by MWU (ns > 0.05, *P ≤ 0.05). (D) Same cells as mentioned in C were subjected to trans‐well invasion assay using Matrigel to cover the inserts. Invaded cells were PFA‐fixed and stained with crystal violet. Pictures were quantified using ImageJ software. Five pictures of each well were calculated. Each data point represents the mean of two replicates from one experiment. Error bar represents the SD of three experiments. Statistical differences between groups were assessed using MWU. Scale μm bar = 400 μm. **P < 0.01.

Overall, we found a new role for DMTF1β in cancer cell motility by regulating ULK1 stability and thereby autophagy induction.

## 4 Discussion

Our studies focused on determining the levels of DMTF1β expression in breast and prostate cancer cells and deciphering the mechanistic basis of DMTF1β's oncogenic role in these cells. Although DMTF1β is expressed at very low levels in cells, we determined that DMTF1β levels were increased in more aggressive breast and prostate cancer cell lines. Previously, we showed that low cellular levels of DMTF1β were sufficient to inhibit DMTF1α‐mediated p14^ARF^ transcription, consistent with DMTF1β having a strong effect at very low cellular concentrations [4, 9]. The low expression and high potency of DMTF1β suggest that it must be tightly regulated to maintain the balance between oncogenic and tumor‐suppressive signaling [4, 9]. Regulation of the DMTF1α/β ratio might be achieved through regulating alternative splicing, mRNA and protein stability, the latter being based on the observation that DMTF1β is a short‐lived protein. Future studies are necessary to identify the key regulators of the DMTF1β oncogene. In this study, we identified a novel function of DMTF1β in promoting breast and prostate cancer cell motility. Moreover, DMTF1β‐mediated cell migration and invasion is mediated via activation of autophagy. However, autophagy has been reported to have migration promoting and inhibiting functions dependent on the context [33, 34, 42, 43]. Mechanistically, autophagy supports focal adhesion turnover. The focal adhesion complex is a large macromolecular assembly located at the leading edge of migrating cells that regulates the mechanical force transmission and signaling events in response to ECM adhesion during cell migration [44, 45, 46]. Furthermore, autophagy regulates epithelial–mesenchymal transition (EMT), an important process that supports the migratory phenotype of metastasizing cells. Silencing the essential autophagy genes ATG3 or ATG7 resulted in reduced TGF‐β/Smad‐dependent signaling and attenuated EMT in hepatocellular and lung carcinoma, while induction of autophagy by inhibiting mTOR or ULK2 resulted in increased EMT and migration in lung cancer cells. Moreover, several publications described that E‐cadherin, which inhibits migration and is important for cell adhesion, is degraded via the autophagic pathway [47, 48, 49, 50, 51]. Of interest, we observed increased expression of the epithelial marker E‐cadherin in DMTF1β knockdown compared to control cells.

DMTF1β is predominantly found in the nucleus suggesting that DMTF1β transcriptionally regulates autophagy‐related genes. Indeed, we found reduced mRNA levels of various autophagy‐related genes when DMTF1β was downregulated in PC3MPRO4 and MDA‐MB‐231 cells. Given DMTF1β lacks most of the DMTF1α DNA binding domain [9, 52, 53], DMTF1β likely does not directly bind to the promoter of these genes, but rather functions as a coregulator recruiting transcriptional regulators to the promoters of autophagy genes. Importantly, although the p53 tumor suppressor can activate transcription of autophagy genes [54, 55, 56], we propose that DMTF1β‐induced autophagy is independent of p53, since our cell line models express mutant p53 and knocking down wildtype p53 had no effect on autophagy or migration of MCF7 breast cancer cells. We identified several novel DMTF1β protein interaction partners in the autophagy pathway, including proteins of the initiation complex such as ULK1 and ATG13. This is initially surprising, since DMTF1β is mainly expressed in the nucleus and autophagy takes place in the cytoplasm [18, 52, 57]. In the present study, we identified that DMTF1β interacts directly or indirectly with ULK1 protein and supports its stability. We speculate that DMTF1β regulates autophagy via stabilization of ULK1, which functions as a positive regulator of basal autophagy. Supporting this notion, overexpression of Beclin‐1, a key downstream target of ULK1 in autophagy, can restore autophagic flux as well as migration in DMTF1β‐depleted cells. The fact that DMTF1β also pulled down ATG13 suggests that DMTF1β is a transient partner of the ULK1 complex. It is noteworthy that DMTF1β may also influence the ULK1 complex assembly. Interestingly, deletion of the β‐specific domain significantly reduced its interaction with ULK1 and ATG13, suggesting that this domain plays a vital role in autophagy. Thus, the mechanism by which DMTF1β stabilizes ULK1 protein, and the function of the β‐domain clearly needs further investigation.

## 5 Conclusion

In conclusion, this study suggests that DMTF1β promotes breast and prostate cancer cell motility by inducing autophagy. We also reveal a novel role of DMTF1β in acting as a critical regulator of ULK1 protein stability. Thus, the DMTF1β–ULK1 axis may be a promising target in cancer.

## Conflict of interest

The authors declare no conflicts of interest.

## Author contributions

JX, NJN, AMS, IT, MH, and DM performed experimental research. FM performed zebrafish experiments. BV and RR analyzed and interpreted the OMICs data; NJN, AMS, and JX drafted the article. JD, BET, IZ, MKJ, and MPT designed the project, wrote the manuscript and gave final approval of the submitted manuscript.

## Supporting information


**Fig. S1.** Validation of DMTF1 splice variant specific primers and probes. (A) DMTF1 isoform expression plasmids were titrated to test the specificity and sensitivity of the β specific primers and probes by qPCR.

**Fig. S2.** Cell proliferation and E‐cadherin (CDH1) expression in DMTF1β‐depleted cancer cells. (A) MCF‐7 control (Ctrl) or DMTF1 knockout clones (KO cl.1 and cl.2) with or without DMTF1β rescue (β) were seeded, and proliferation was assessed via TrypanBlue exclusion cell counting at 24 h and 48 h. Analyses were performed using Prism GraphPad software. Data are shown as Mean ± SD from four independent experiments with two replicates each. Differences to control cells (ctrl) was assessed by TWA followed by Dunnett's multiple comparisons test (ns, not significant). (B) DMTF1β mRNA is shown with the location of the designed shRNAs targeting the β isoform. The shRNAs target intron 9 of the DMTF1 gene, a region specific to the DMTF1β isoform. Lines indicate the positions of the shRNAs. Images were created with BioRender.com. (C) Experiments as in S2B using MDA‐MB‐231 and PC3MPRO4 DMTF1β (shβ_1 and _2) knockdown and control cells (shCtrl). (D) A repeat Western Blots of DMTF1β protein levels in MDA‐MB‐231 and PC3MPRO4 control (shCtrl) and DMTF1β knockdown (shβ_1, shβ_2) cells are shown. Quantification was performed using imagej and normalized to the total protein and the protein level in the respective shCtrl cell line. (E) *CDH1* mRNA levels cells in S2B and S2C were quantified by RT‐qPCR. mRNA levels were normalized to the housekeeping gene hydroxymethylbilane synthase (*HMBS*) and are shown as n‐fold expression compared to control cells. Error bars represent standard deviation from 3 independent experiments in duplicates. (MWU, **P < 0.01).

**Fig. S3.** WIPI1 and VPS34 levels in DMTF1β depleted cells. (A) Western Blot analysis of PC3MPRO4 cells expressing control shRNA (shCtrl) or DMTF1β‐targeting shRNA (shβ_2), with or without DMTF1β–HiBiT (β‐HiBiT) or β domain–deleted mutant (DMTF1ΔMHR–HiBiT), is shown. Representative Western Blot of two independent experiments (Rep1 and 2) showing the protein levels of VPS34 and WIPI1. Total protein is used as loading control. (B) Graphs represent mean ± SD of four or five independent experiments described in A. Band intensities were determined using imagej. Protein levels were normalized to total protein. One‐Way ANOVA followed by Holm‐Šídák's multiple comparisons test was applied to asses’ differences between selected groups (ns, not significant).

**Fig. S4.** Autophagic flux and cell migration in DMTF1β or p53 depleted cancer cells. (A) PC3MPRO4 and MDA‐MB‐231 DMTF1β (shβ_1 and shβ_2) knockdown or control shRNA (shCtrl) cells ectopically expressing the HyD‐LIR(TP)‐GFP construct were treated with DMSO or Bafilomycin A1 (Baf. A1) for 2 h. Cells were fixed and analyzed using a confocal microscope. The number of GFP positive dots per cell were quantified and the autophagic flux calculated by subtracting the number of dots in the absence of Baf. A1 from the number of dots in presence of Baf. A1‐Graph show mean ± SD (MWU, ***P < 0.001; ****P < 0.0001 from three independent experiments). (B) Wildtype p53 was silenced using two different siRNAs in MCF7 control, DMTF1β KO (KO1) and DMTF1β reconstituted DMTF1 KO cells. A non‐targeting scrambled siRNA (siCtrl) was used as control. p53 protein levels and autophagy were measured by western blotting of p53 and LC3B, respectively. The autophagic flux was determined by subtracting the amount of lipidated LC3B (LC3B‐II) in Baf. A1 from the amount in DMSO treated cells. Experiment was carried out 4 times. Error bars represent SD (MWU, ns, not significant). (C) Wound healing assays of cells described in S3B. Migration was assessed by using Cell‐IQ live cell imaging. Images were taken every hour of the same wound area and wound closure was measured by Activision software. TWA followed by Dunnett's multiple comparison test was used to assess significance between groups (ns, not significant).

**Fig. S5.** DMTF1β binds and modulates ULK1 protein levels A–B Western Blots of the second of the two co‐IPs performed. Details are described in Figure 6A or 6B, respectively. (C) The amounts of ULK1 and ATG13 detected in the Co‐immunoprecipitation (Co‐IP) samples were quantified and normalized to the corresponding Flag–β or Flag–ΔMHR signals within the same IP fractions from two independent co‐IPs. (D) Schematic overview of Cas9‐gRNA mediated HiBiT knock‐in at the DMTF1β C‐terminus in MCF7 cells and subsequent downstream applications. IF, immunofluorescence; PLA, proximity ligation assay; WB, Western Blot. (E) MCF7 cells harboring a knock‐in of HiBiT at the C‐terminus of DMTF1β were assessed to luminescence measurements after the addition of Nano‐Glo® HiBiT Lytic reagent (Promega). MCF7 parentals were used as a negative control. (F) MCF7 with endogenous HiBiT‐tagged DMTF1β were infected with a control or an shRNA to target DMTF1β and luminescence was measured as described in E. (G, H) MCF‐7 control cells or MCF‐7 cells with endogenously HiBiT‐tagged DMTF1β were treated with Torin1 or vehicle control (DMSO) for 2 h, followed by proximity ligation assay (PLA) to detect ULK1–HiBiT interactions. (G) Representative images showing ULK1–HiBiT interactions detected by PLA. Scale bar indicates 25 μm. (H) PLA signals were quantified as the number of dots per field of view (FoV) from three independent experiments, with four images analyzed per experiment. Dot counts were normalized to the number of cells per image. Statistical significance between groups was assessed using two‐way ANOVA. Data are presented as mean ± SD; **** indicates *P* < 0.0001. (I) Western Blot for ULK1 and ATG13 in PC3MPRO4 cells expressing either control or shRNAs targeting DMTF1β (shβ_1 or_2). (J) Mean expression levels of ULK1 and ATG13 in PC3MPRO4 cells expressing either control or shRNAs targeting DMTF1β (shβ_1 or_2). Error bars show SD from 4 independent experiments. Protein levels were normalized to total protein and shCtrl of each experiment. Kruskal‐Wallis followed by Dunn's multiple comparison test was used to compare the groups (ns, not significant, *P ≤ 0.05). (K) ULK1 mRNA levels were assessed by qPCR in PC3MPRO4 cells depleted or not for DMTF1β. Mean ± standard deviation from three independent experiments is shown. MWU was applied to test for significance (ns, not significant).

**Fig. S6.** DMTF1β‐mediated autophagy drives cancer cell migration. (A, B) Autophagic activity was assessed in MCF‐7 cells stably expressing the LC3B‐mCherry‐GFP tandem construct. Cells were treated for 24 h with the autophagy inhibitors (VPS34‐IN1 or MRT68921) at indicated concentrations for 24 h before autophagic activity was assessed by mCherry/GFP ratiometric FACS analysis. Data was analyzed by FlowJo software. Error bars represent mean ± SD of three independent experiments in duplicates. (MWU; *P ≤ 0.05). (C) Migration was assessed by wound closure assay in MCF‐7 control (KO Ctrl) and DMTF1 knock‐out (KO^DMTF1^ cl.1) clones ± DMTF1β rescue (KO^DMTF1^ cl.1/DMTF1β) using Cell‐IQ live cell imaging. Autophagy was blocked using VPS34IN1. Images were taken every hour of the same wound area and wound closure was measured by Activision software. TWA followed by Dunnett's multiple comparison test was used to assess significance between groups from four independent experiments (ns, not significant, *P ≤ 0.05, **P < 0.01). (D, E) ULK1 and VPS34 were downregulated via stable expression of gene specific shRNAs (sh*ULK1*, sh*VPS34*) in MCF7 control, DMTF1 KO (KO cl. 1) and DMTF1β reconstituted DMTF1 KO cells. Knockdown efficiency was assessed performing ULK1 and VPS34 western blotting from two independent experiments. Autophagy was measured by LC3B western blotting. The autophagic flux was determined by the amount of lipidated LC3B (LC3B‐II) in Baf. A1 treated samples subtracted by the amount of LC3B‐II in DMSO treated cells. Error bars are from four independent experiments. Analyses were performed using imagej software MWU; ns, not significant, *P ≤ 0.05, **P < 0.01.

**Table S1.** Differential gene expression analysis in control and DMTF1β‐knockdown PC3MPro4 cells based on RNA sequencing.

## Data Availability

The datasets used and analyzed in this study are included in this published article and its supplementary information files.
