# Supplementary material for: Oncogenic DMTF1β promotes cancer cell motility by regulating autophagy through ULK1 stabilization
Source: Mol Oncol. 2026 May 26;20(11):2718–40. doi: 10.1002/1878-0261.70275 (PMC13398728; doi:10.1002/1878-0261.70275)

Supplementary Figure 5

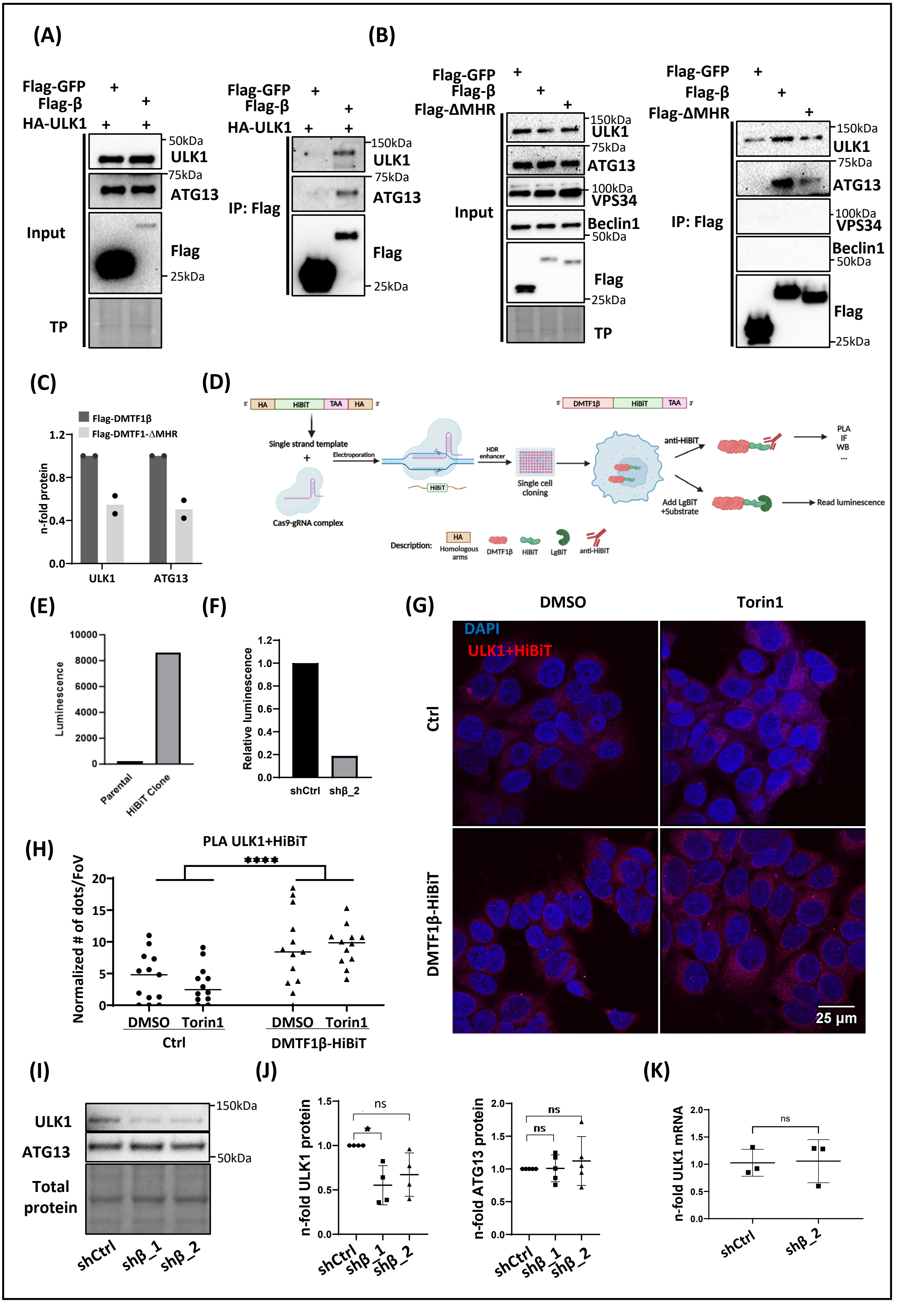

Supplement: Supplementary file 3 [file MOL2-20-2718-s003.pdf]
