# Supplementary figures and images for: Oncogenic DMTF1β promotes cancer cell motility by regulating autophagy through ULK1 stabilization
Source: Mol Oncol. 2026 May 26:10.1002/1878-0261.70275. Online ahead of print. doi: 10.1002/1878-0261.70275 (PMC13398728; doi:10.1002/1878-0261.70275)

Supplementary Figure 1

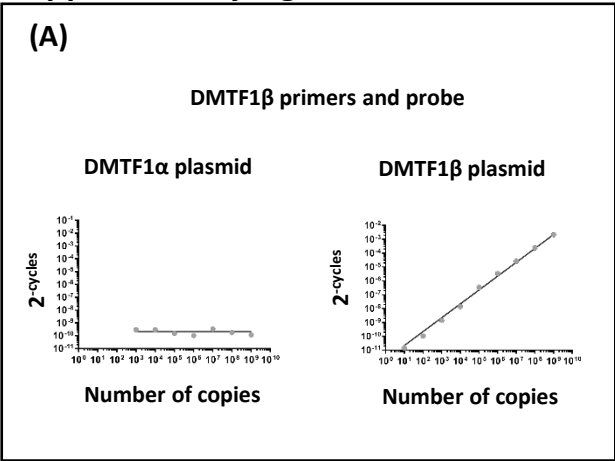

Supplement: Supplementary file 1 — Fig. S1. Validation of DMTF1 splice variant specific primers and probes. (A) DMTF1 isoform expression plasmids were titrated to test the specificity and sensitivity of the β specific primers and probes by qPCR. [file MOL2-9999-0-s003.pdf]

Supplementary Figure 2

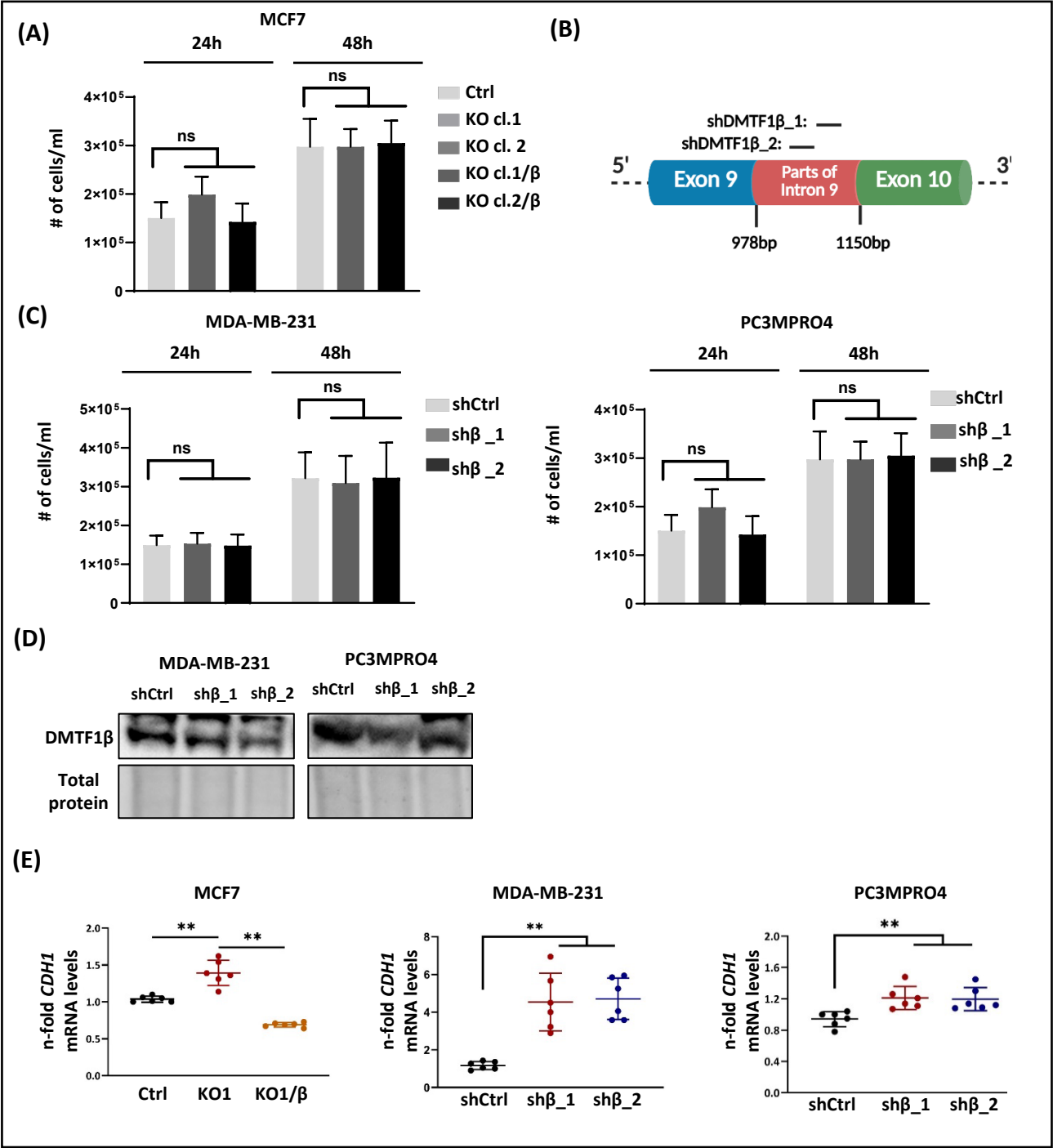

Supplement: Supplementary file 2 — Fig. S2. Cell proliferation and E‐cadherin (CDH1) expression in DMTF1β‐depleted cancer cells. (A) MCF‐7 control (Ctrl) or DMTF1 knockout clones (KO cl.1 and cl.2) with or without DMTF1β rescue (β) were seeded, and proliferation was assessed via TrypanBlue exclusion cell counting at 24 h and 48 h. Analyses were performed using Prism GraphPad software. Data are shown as Mean ± SD from four independent experiments with two replicates each. Differences to control cells (ctrl) was assessed by TWA followed by Dunnett's multiple comparisons test (ns, not significant). (B) DMTF1β mRNA is shown with the location of the designed shRNAs targeting the β isoform. The shRNAs target intron 9 of the DMTF1 gene, a region specific to the DMTF1β isoform. Lines indicate the positions of the shRNAs. Images were created with BioRender.com. (C) Experiments as in S2B using MDA‐MB‐231 and PC3MPRO4 DMTF1β (shβ_1 and _2) knockdown and control cells (shCtrl). (D) A repeat Western Blots of DMTF1β protein levels in MDA‐MB‐231 and PC3MPRO4 control (shCtrl) and DMTF1β knockdown (shβ_1, shβ_2) cells are shown. Quantification was performed using imagej and normalized to the total protein and the protein level in the respective shCtrl cell line. (E) CDH1 mRNA levels cells in S2B and S2C were quantified by RT‐qPCR. mRNA levels were normalized to the housekeeping gene hydroxymethylbilane synthase (HMBS) and are shown as n‐fold expression compared to control cells. Error bars represent standard deviation from 3 independent experiments in duplicates. (MWU, **P < 0.01). [file MOL2-9999-0-s004.pdf]

Supplementary Figure 3

(A)

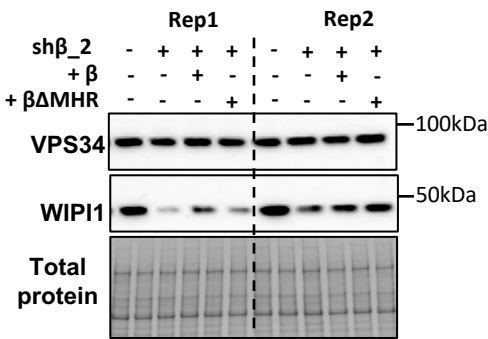

(B)

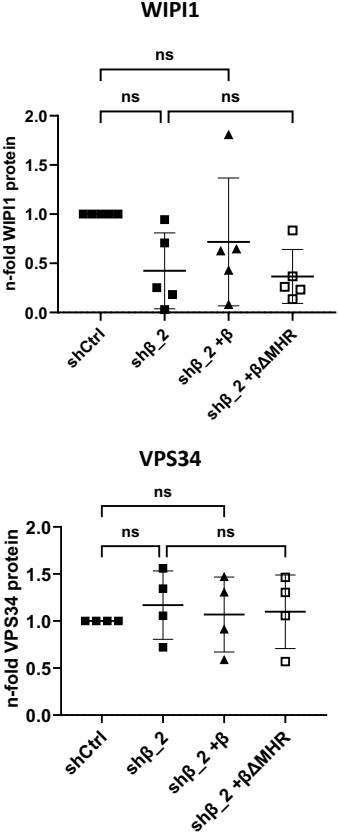

Supplement: Supplementary file 3 — Fig. S3. WIPI1 and VPS34 levels in DMTF1β depleted cells. (A) Western Blot analysis of PC3MPRO4 cells expressing control shRNA (shCtrl) or DMTF1β‐targeting shRNA (shβ_2), with or without DMTF1β–HiBiT (β‐HiBiT) or β domain–deleted mutant (DMTF1ΔMHR–HiBiT), is shown. Representative Western Blot of two independent experiments (Rep1 and 2) showing the protein levels of VPS34 and WIPI1. Total protein is used as loading control. (B) Graphs represent mean ± SD of four or five independent experiments described in A. Band intensities were determined using imagej. Protein levels were normalized to total protein. One‐Way ANOVA followed by Holm‐Šídák's multiple comparisons test was applied to asses’ differences between selected groups (ns, not significant). [file MOL2-9999-0-s007.pdf]

Supplementary Figure 4

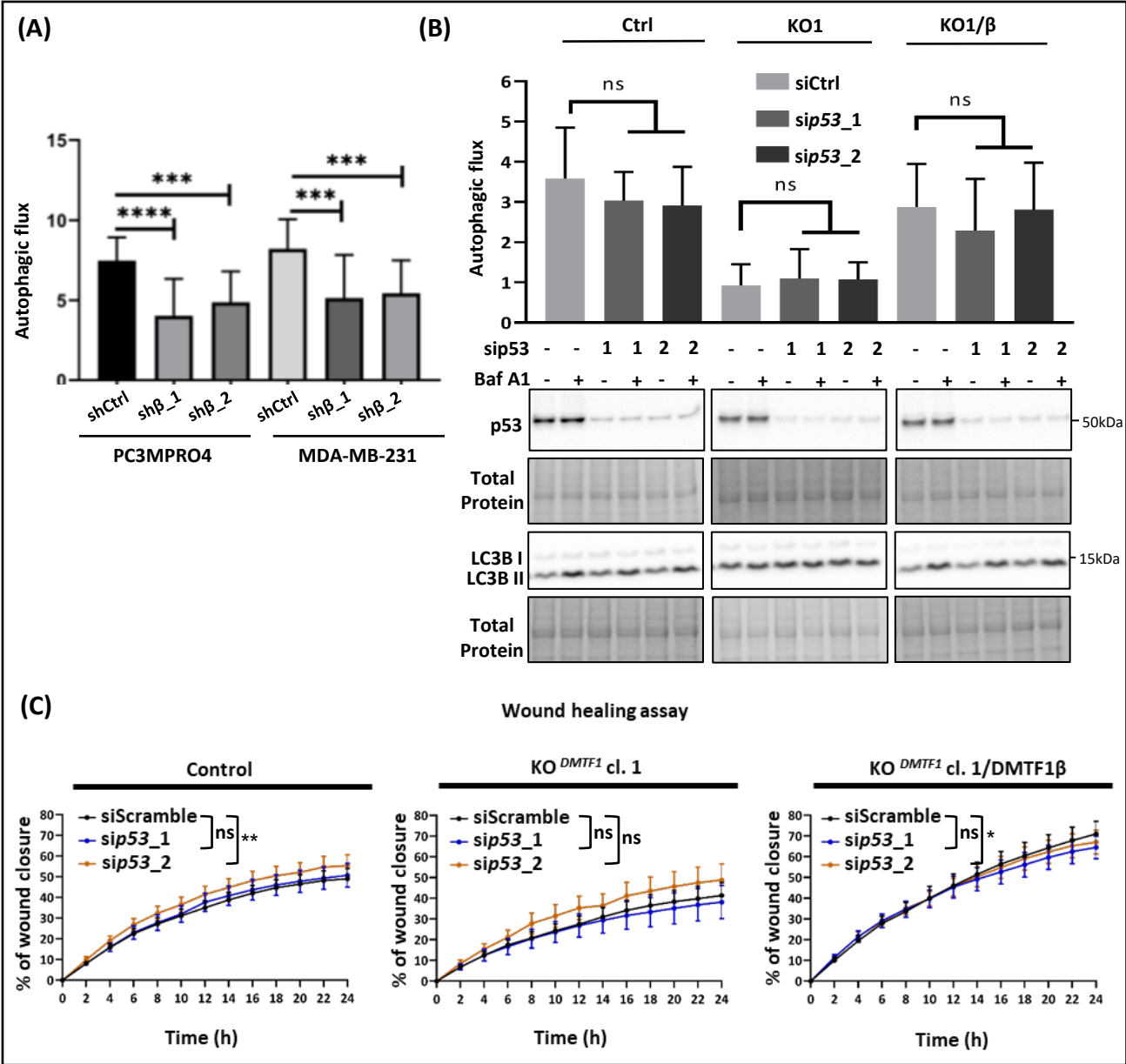

Supplement: Supplementary file 4 — Fig. S4. Autophagic flux and cell migration in DMTF1β or p53 depleted cancer cells. (A) PC3MPRO4 and MDA‐MB‐231 DMTF1β (shβ_1 and shβ_2) knockdown or control shRNA (shCtrl) cells ectopically expressing the HyD‐LIR(TP)‐GFP construct were treated with DMSO or Bafilomycin A1 (Baf. A1) for 2 h. Cells were fixed and analyzed using a confocal microscope. The number of GFP positive dots per cell were quantified and the autophagic flux calculated by subtracting the number of dots in the absence of Baf. A1 from the number of dots in presence of Baf. A1‐Graph show mean ± SD (MWU, ***P < 0.001; ****P < 0.0001 from three independent experiments). (B) Wildtype p53 was silenced using two different siRNAs in MCF7 control, DMTF1β KO (KO1) and DMTF1β reconstituted DMTF1 KO cells. A non‐targeting scrambled siRNA (siCtrl) was used as control. p53 protein levels and autophagy were measured by western blotting of p53 and LC3B, respectively. The autophagic flux was determined by subtracting the amount of lipidated LC3B (LC3B‐II) in Baf. A1 from the amount in DMSO treated cells. Experiment was carried out 4 times. Error bars represent SD (MWU, ns, not significant). (C) Wound healing assays of cells described in S3B. Migration was assessed by using Cell‐IQ live cell imaging. Images were taken every hour of the same wound area and wound closure was measured by Activision software. TWA followed by Dunnett's multiple comparison test was used to assess significance between groups (ns, not significant). [file MOL2-9999-0-s002.pdf]

Supplementary Figure 5

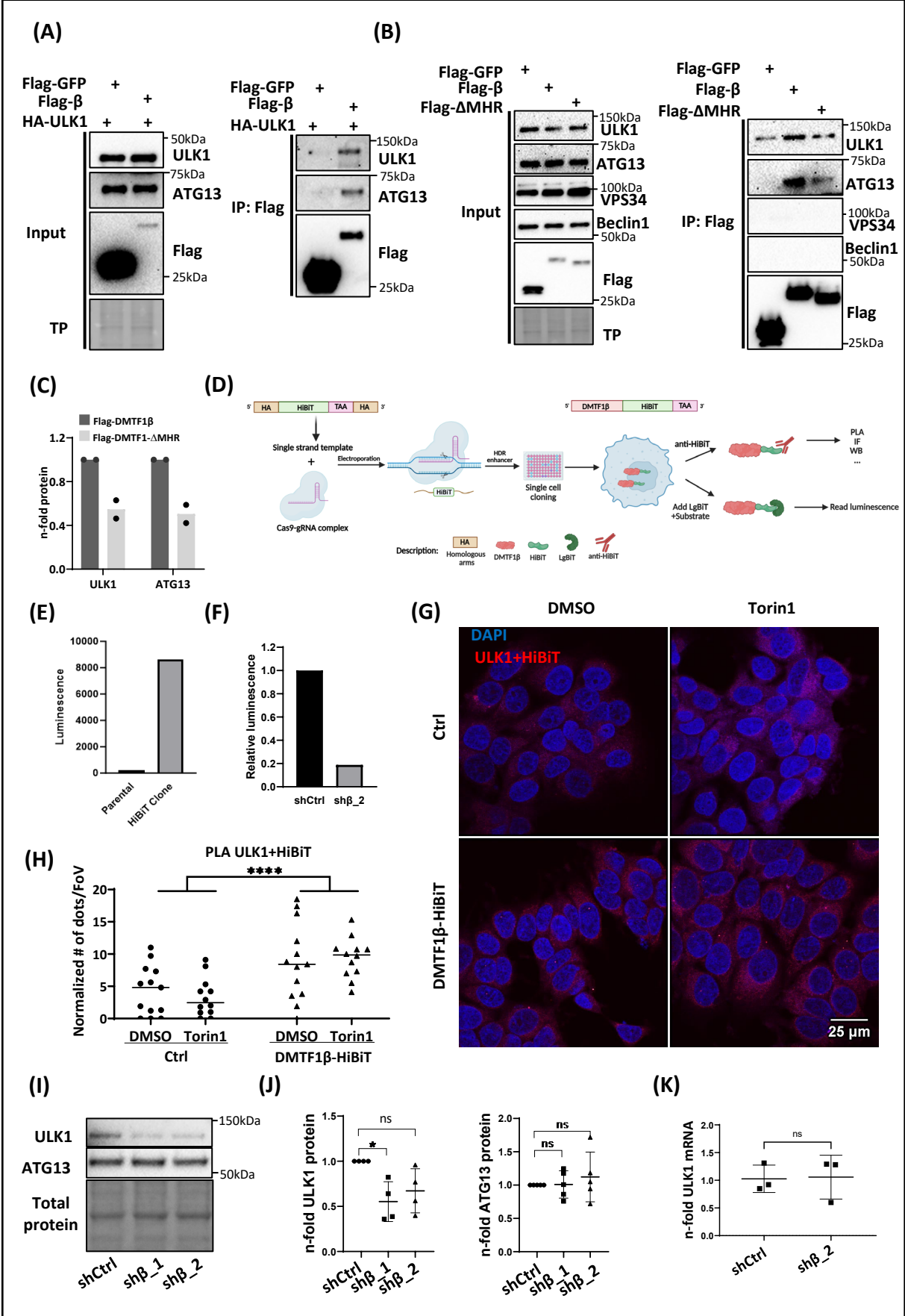

Supplement: Supplementary file 5 — Fig. S5. DMTF1β binds and modulates ULK1 protein levels A–B Western Blots of the second of the two co‐IPs performed. Details are described in Figure 6A or 6B, respectively. (C) The amounts of ULK1 and ATG13 detected in the Co‐immunoprecipitation (Co‐IP) samples were quantified and normalized to the corresponding Flag–β or Flag–ΔMHR signals within the same IP fractions from two independent co‐IPs. (D) Schematic overview of Cas9‐gRNA mediated HiBiT knock‐in at the DMTF1β C‐terminus in MCF7 cells and subsequent downstream applications. IF, immunofluorescence; PLA, proximity ligation assay; WB, Western Blot. (E) MCF7 cells harboring a knock‐in of HiBiT at the C‐terminus of DMTF1β were assessed to luminescence measurements after the addition of Nano‐Glo® HiBiT Lytic reagent (Promega). MCF7 parentals were used as a negative control. (F) MCF7 with endogenous HiBiT‐tagged DMTF1β were infected with a control or an shRNA to target DMTF1β and luminescence was measured as described in E. (G, H) MCF‐7 control cells or MCF‐7 cells with endogenously HiBiT‐tagged DMTF1β were treated with Torin1 or vehicle control (DMSO) for 2 h, followed by proximity ligation assay (PLA) to detect ULK1–HiBiT interactions. (G) Representative images showing ULK1–HiBiT interactions detected by PLA. Scale bar indicates 25 μm. (H) PLA signals were quantified as the number of dots per field of view (FoV) from three independent experiments, with four images analyzed per experiment. Dot counts were normalized to the number of cells per image. Statistical significance between groups was assessed using two‐way ANOVA. Data are presented as mean ± SD; **** indicates P < 0.0001. (I) Western Blot for ULK1 and ATG13 in PC3MPRO4 cells expressing either control or shRNAs targeting DMTF1β (shβ_1 or_2). (J) Mean expression levels of ULK1 and ATG13 in PC3MPRO4 cells expressing either control or shRNAs targeting DMTF1β (shβ_1 or_2). Error bars show SD from 4 independent experiments. Protein levels were normalized to to [file MOL2-9999-0-s006.pdf]

Supplementary Figure 6

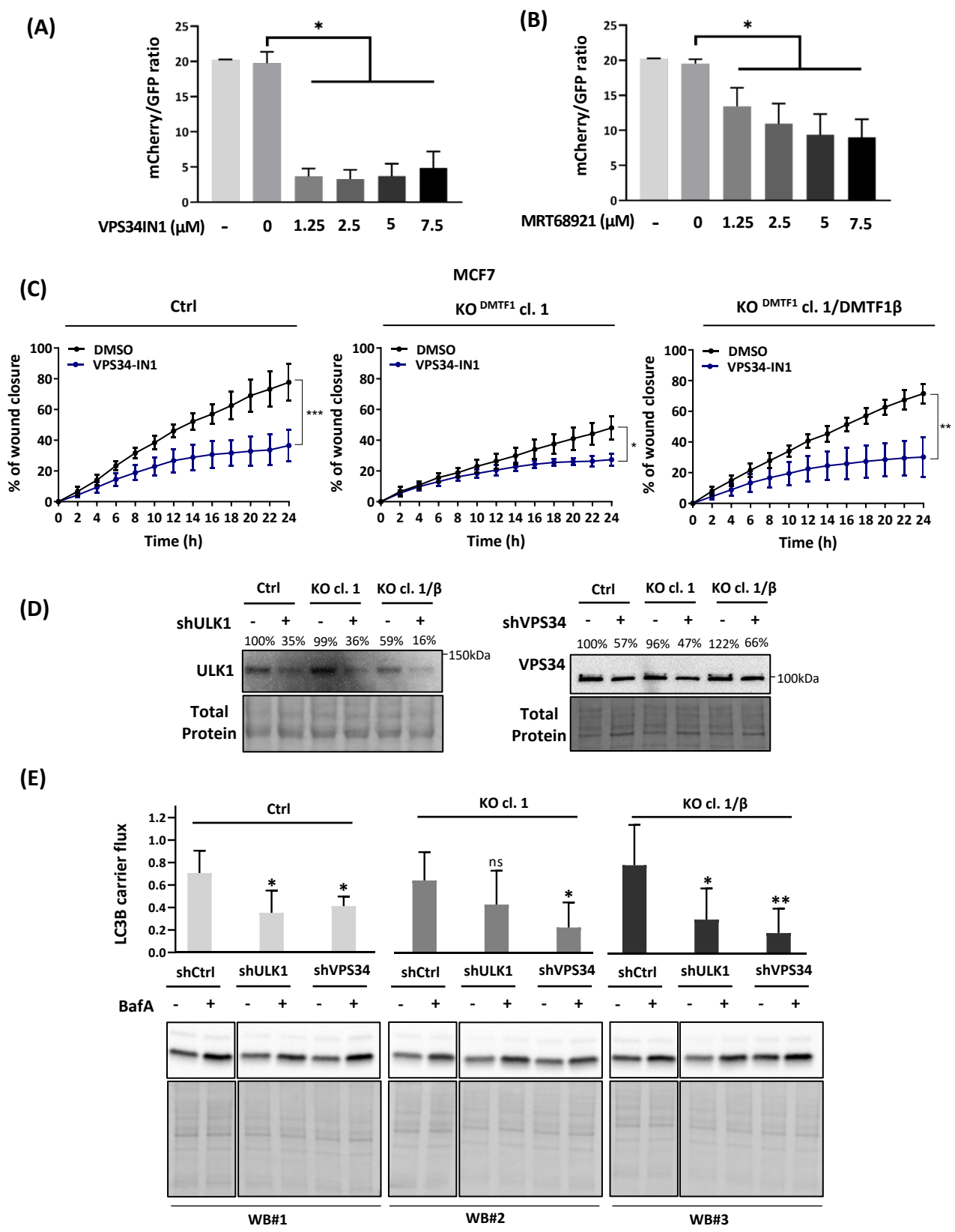

Supplement: Supplementary file 6 — Fig. S6. DMTF1β‐mediated autophagy drives cancer cell migration. (A, B) Autophagic activity was assessed in MCF‐7 cells stably expressing the LC3B‐mCherry‐GFP tandem construct. Cells were treated for 24 h with the autophagy inhibitors (VPS34‐IN1 or MRT68921) at indicated concentrations for 24 h before autophagic activity was assessed by mCherry/GFP ratiometric FACS analysis. Data was analyzed by FlowJo software. Error bars represent mean ± SD of three independent experiments in duplicates. (MWU; *P ≤ 0.05). (C) Migration was assessed by wound closure assay in MCF‐7 control (KO Ctrl) and DMTF1 knock‐out (KODMTF1 cl.1) clones ± DMTF1β rescue (KODMTF1 cl.1/DMTF1β) using Cell‐IQ live cell imaging. Autophagy was blocked using VPS34IN1. Images were taken every hour of the same wound area and wound closure was measured by Activision software. TWA followed by Dunnett's multiple comparison test was used to assess significance between groups from four independent experiments (ns, not significant, *P ≤ 0.05, **P < 0.01). (D, E) ULK1 and VPS34 were downregulated via stable expression of gene specific shRNAs (shULK1, shVPS34) in MCF7 control, DMTF1 KO (KO cl. 1) and DMTF1β reconstituted DMTF1 KO cells. Knockdown efficiency was assessed performing ULK1 and VPS34 western blotting from two independent experiments. Autophagy was measured by LC3B western blotting. The autophagic flux was determined by the amount of lipidated LC3B (LC3B‐II) in Baf. A1 treated samples subtracted by the amount of LC3B‐II in DMSO treated cells. Error bars are from four independent experiments. Analyses were performed using imagej software MWU; ns, not significant, *P ≤ 0.05, **P < 0.01. [file MOL2-9999-0-s005.pdf]
